# Return-to-work for people living with long COVID: A scoping review of interventions and recommendations

**DOI:** 10.1371/journal.pone.0321891

**Published:** 2025-10-15

**Authors:** Gagan Nagra, Victor E. Ezeugwu, Geoff P. Bostick, Erin Branton, Liz Dennett, Kevin Drake, Quentin Durand-Moreau, Christine Guptill, Mark Hall, Chester Ho, Pam Hung, Aiza Khan, Grace Y. Lam, Behdin Nowrouzi-Kia, Douglas P. Gross

**Affiliations:** 1 Rehabilitation Science Program, Faculty of Rehabilitation Medicine, University of Alberta, Edmonton, Canada; 2 Department of Physical Therapy, Faculty of Rehabilitation Medicine, University of Alberta, Edmonton, Canada; 3 Geoffrey and Robyn Sperber Health Sciences Library, University of Alberta, Edmonton, Canada; 4 Service Manager, Workers' Compensation Board Alberta Millard Health, Edmonton, Canada; 5 Division of Preventive Medicine, Faculty of Medicine and Dentistry, University of Alberta, Edmonton,; 6 School of Rehabilitation Sciences, University of Ottawa, Ottawa, Canada; 7 Department of Medicine, Faculty of Medicine & Dentistry, University of Alberta, Edmonton, Canada; 8 Department of Medicine, Division of Pulmonary Medicine, University of Alberta, Edmonton,; 9 Department of Occupational Science and Occupational Therapy, Temerity Faculty of Medicine, University of Toronto, Ontario, Canada; Mayo Clinic College of Medicine and Science, UNITED STATES OF AMERICA

## Abstract

**Introduction:**

Long COVID is characterized by the presence of new onset or persistent symptoms 3 months after a suspected or confirmed history of SARS-CoV-2 infection. It is a complex and multi-faceted condition that affects people in different ways. Long COVID affects individuals’ labour market participation. While some cannot work, others may return to work (RTW) in a limited capacity. Determining what rehabilitation or related strategies are safe and effective for facilitating RTW is necessary.

**Objectives:**

To synthesize evidence on RTW interventions for people living with Long COVID and to identify ‘promising’ interventions for enhancing work ability and RTW.

**Methods:**

We followed Arksey & O’Malley’s methodology and the PRISMA extension for scoping reviews. Five electronic bibliographic databases and grey literature were searched. The literature search included various study designs, such as randomized controlled trials (RCT), quasi-experimental designs, and observational studies as well as clinical practice guidelines (CPGs). Two reviewers conducted screening and data extraction, with disagreements resolved through consensus. Intervention studies were categorized as promising (statistically significant RTW outcomes or ≥ 50% RTW), somewhat promising (20% to < 50% RTW), not promising (non-statistically significant RTW outcomes or < 20% RTW), or uncertain (did not specify proportion of RTW).

**Results:**

Twelve CPGs and nineteen intervention studies were identified. Of the intervention studies, 5 were cohort studies, 3 quasi-experimental studies, 4 observational, 2 interventional, 3 RCTs, and 2 case reports. Promising interventions included multimodal and interdisciplinary work-focused rehabilitation, multidisciplinary inpatient and outpatient rehabilitation, psychoeducation, pacing, and breathing strategies, shifting focus from symptom monitoring to optimizing functional outcomes, enhanced external counterpulsation inflatable pressure to improve blood flow, and constraint-induced cognitive therapy.

**Conclusion:**

Many uncertainties remain regarding which RTW interventions are effective or the optimal characteristics of these interventions.

## 1. Background

Long COVID is characterized by the presence of new onset or persistent symptoms 3 months after a suspected or confirmed history of SARS-CoV-2 infection [1,2]. Long COVID is a complex and multi-faceted condition that affects individuals in different ways [3]. About 16% of Canadians infected with COVID-19 developed Long COVID, with higher prevalence in women, individuals hospitalized due to COVID-19, those with pre-existing health conditions, and those with repeated COVID-19 infections [2]. Long COVID symptoms limit daily functioning, workability, and employment, with up to 76% of people living with Long COVID taking time off work or entirely stopping work [2,4–6]. Previous qualitative research highlighted significant challenges faced by individuals with Long COVID in performing daily activities and meaningful work-related tasks [7,8]. About 3.5 million Canadian adults reported experiencing long-term symptoms following a COVID-19 infection with about 100,000 reporting they were still off work due to symptoms as of June 2024. [2,9] This corresponds to approximately 0.45% of the Canadian labor force.

Systemic exertion intolerance, characterized by severe fatigue worsened by physical or mental exertion, is a prevalent symptom in Long COVID and can severely impact sustained work activity [1,4,10]. Factors like exercise, stress, cognitive tasks, and sleep deprivation can exacerbate symptoms of systemic exertion intolerance [2,4]. This prevalent symptom is similar to Myalgic Encephalomyelitis (ME), and some individuals with Long COVID meet diagnostic criteria for ME [1,10–12]. Other challenges faced by people living with Long COVID include fluctuating and episodic symptoms, sleep disruption, and fatigue, which hinder daily activities and return to work (RTW) [2]. Traditional therapies like graded exercise may not be effective or could be harmful for some people living with Long COVID, particularly in those experiencing Post-Exertional Malaise (PEM) [13]. This necessitates exploring new strategies. Despite recognition of the burden of Long COVID, research on strategies to improve daily function and workability is limited.

Rehabilitation is one option for potentially addressing Long COVID activity limitations and participation restrictions. Rehabilitation aims to decrease the burden of physical, neurocognitive, and psychological limitations by stabilizing or enhancing patients’ physical and mental abilities [14]. However, there are few Long COVID-specific rehabilitation tools or protocols to address various symptoms such as fatigue or cognitive dysfunction [15]. This can lead to diverse and inconsistent intervention approaches or even potentially harmful solutions such as graded exercise, which is now contraindicated for those experiencing PEM [2,15,16]. Research is still inconclusive regarding other exercise-based rehabilitation interventions [6,15,16].

Determining what rehabilitation strategies are safe and effective for facilitating RTW is necessary. There is an urgent need for safe, effective and evidence-based Long COVID treatment programs [15]. Some studies of occupational rehabilitation strategies have been published, although the literature is diverse, emerging and requires synthesis [7,14,17,18]. A comprehensive synthesis would help to inform global clinical practice guidelines. The purpose of this scoping review is to 1) synthesize evidence on interventions used to promote return-to-work or enhance work ability among people living with Long COVID and 2) identify the most promising interventions for enhancing work ability and RTW.

## 2. Methods

### Study design

For this scoping review, the Arksey & O’Malley methodological framework was applied and reported based on the PRISMA extension for scoping studies [19]. This methodological framework consists of the following stages: identifying the research question, identifying relevant studies, study selection, charting the data, collating, summarizing, and reporting results, and consultation (optional stage) [19].

We followed the PI(E)COS framework (population, intervention (exposure), comparators, outcomes, study design/setting) and included various study designs, such as randomized controlled trials, quasi-experimental designs, and observational studies. We also included clinical practice guidelines that had relevant recommendations regarding RTW.

### Search terms and search strategies

Members of the research team met with a health sciences librarian (LD) to perform preliminary searches and develop an extensive list of relevant terms. The librarian then conducted a systematic search of MEDLINE (via Ovid), Embase (via Ovid), APA PsycINFO (via Ovid), CINAHL Plus with Full Text (via EBSCOhost), Scopus, and Cochrane Library CENTRAL trials (via Wiley) from database inception until January 3, 2025. The search strategy included a combination of subject headings and keywords to combine the concepts of Long COVID and either occupational rehabilitation or occupational functioning/outcomes. The search was optimized for running in each database. No date, language or publication type limits were applied to the search.

In addition to searching academic databases for peer reviewed literature, an extensive grey literature search was conducted. The librarian searched up to January 3, 2025 the following websites and online databases: MedRxiv (https://www.medrxiv.org/), Bielefeld Academic Search Engine (https://www.base-search.net/), OAIster (oaister.on.worldcat.org), Custom Google Search Engine for Canadian Public Health Information (https://www.ophla.ca/p/customsearchcanada.html), Custom Google Search Engine for US State Government Information (https://www.ophla.ca/p/customsearchusstates.html), Government of Canada Publications (https://www.publications.gc.ca/site/eng/home.html), Alberta Health Services Long COVID page (https://www.albertahealthservices.ca/topics/Page17540.aspx), WHO pages relevant to Long COVID and a number of general searches on Google. The full search strategy is available in the Supplemental Appendix.

### Selecting studies for analysis.

The following was the final set of inclusion/exclusion criteria for the review:

*Topic of the article* – Evaluation of an intervention aimed at increasing work functioning or workability.

*Population* – People living with Long COVID. Our review included people with a variety of symptoms and activity limitations due to Long COVID. Given the diverse nomenclature in this area, a variety of search terms were included.

*Intervention* – We focused primarily on studies evaluating interventions aimed at improving work functioning or promoting RTW. From the perspective of the various partners involved (i.e. people living with Long COVID, workers’ compensation insurers, employers and health care providers), work-related functional recovery — such that the patient can return to sustainable and predictable work activity — is important and has important career and quality-of-life implications.

*Outcome* – Work-related outcomes, including RTW, stay at work, work disability, work absence/absenteeism, and work productivity or related constructs (i.e., presenteeism, etc.).

*Study type* – Any design evaluating an intervention for Long COVID. Systematic reviews were excluded but references within those located were searched for further articles.

#### Screening of relevant articles.

The titles and abstracts of articles obtained from the online databases were reviewed and appraised for relevance. Two researchers from the team read each title/abstract independently and judged whether they were relevant to the research question. When there were disagreements between reviewers, the principal researcher (DPG) offered additional consultation until a consensus was reached. If relevance of a study was still unclear, then the full article was obtained. After identifying relevant abstracts and titles, two independent researchers assessed the corresponding full versions of the studies to determine which articles were relevant for inclusion in the full review. All team members used Covidence software (Melbourne, Australia) to organize data at all review stages.

### Consultation with people with lived experiences and knowledge users

The consultation process for this study included a large multidisciplinary team consisting of people with lived experiences (PWLE) of Long COVID impacting workability, researchers from various health professions (occupational medicine, physiotherapy, occupational therapy, and kinesiology), and knowledge users who were experienced clinicians providing rehabilitation for Long COVID or employees of the Workers’ Compensation Board of Alberta. PWLE or delivering health care for Long COVID were actively involved throughout the study, providing input on the proposal and initial questions as well as informing data collection, results interpretation and conclusions. Input was also gathered through a dedicated workshop held at a Long COVID Symposium, where recommendations and insights were shared related to RTW based on lived experiences with Long COVID [20]. This feedback informed our understanding of RTW challenges and contributed to the interpretation of findings. In addition, we drew on guidance from published literature on patient-oriented research [21]. Throughout the project, we sought to engage a diverse group of individuals with an interest in Long COVID and RTW, including PWLE, healthcare and rehabilitation professionals, and workers’ compensation representatives, to inform study selection, intervention categorization, and contextualization of results.

Meetings were held with the full team, then periodic email updates were provided to seek advice on selecting studies for analysis and to summarize and report results. PWLE and knowledge users were asked whether they knew of any interventions currently in use and found to be safe and effective for Long COVID. Feedback highlighted the importance of: 1) considering the variable and episodic nature of Long COVID symptoms as a major barrier to RTW; 2) including RTW outcomes as search terms; 3) considering not only papers describing specific interventions, but also theoretical or conceptual papers dealing with models or pathways for people living with Long COVID; and lastly, 4) considering the importance of safety, feasibility, burden, and need for training in addition to scientific validation when considering the interventions identified from the literature search. Before charting the data, PWLE and knowledge users were consulted to determine whether the number of articles selected was appropriate and whether the search terms should be altered.

### Data analysis

#### Charting the data.

Reviewers extracted relevant information from each article. This included the author list, year of publication, article title, geographic location of the study, type and brief description of the intervention, study population, study design and goals, methods used, outcome measures used, important results and any economic data recorded.

#### Collating, summarizing and reporting results.

During this stage, we created an overview of all research located. Initially, we presented a basic numerical summary of the studies, including the extent, nature and distribution of the articles. We then summarized articles according to the types of tools described or evaluated, research methods used, populations studied, and study results/outcomes. Since the scoping review methodology was intended to summarize both the breadth and depth of the literature, we reported the number of articles for various interventions as well as descriptive information about the articles.

We attempted to map the diversity of studies observed to create an inventory of the various study designs and methods used. This procedure allowed us to draw conclusions about the nature of research in this area and provide recommendations for future studies. The various interventions identified in the articles were categorized according to level of intervention promise, and key concepts and terminology used in the articles were summarized. To collate, summarize, and report results for this scoping review, we conducted a descriptive characteristic summary and content analysis of intervention studies [19].

We categorized interventions as promising, somewhat promising, or not promising based on a previously published categorization method [22,23]. Interventions were considered promising when results demonstrated statistically significant RTW outcomes or ≥ 50% RTW success. We categorized interventions as somewhat promising when RTW outcomes ranged from 20% to < 50% RTW success. We categorized interventions as not promising when results demonstrated non-statistically significant RTW outcomes or < 20% RTW success. Studies that did not examine RTW rates specifically, but examined other aspects of work ability were rated as ‘uncertain’.

## 3. Results

**Fig 1:**
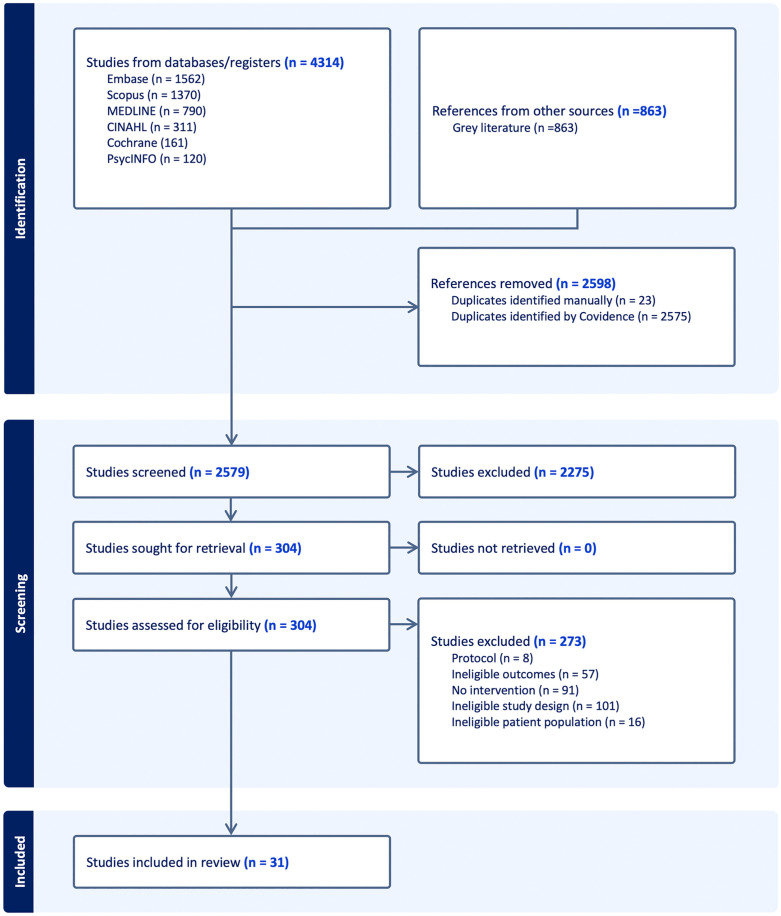
PRISMA flow diagram.

**Fig 2:**
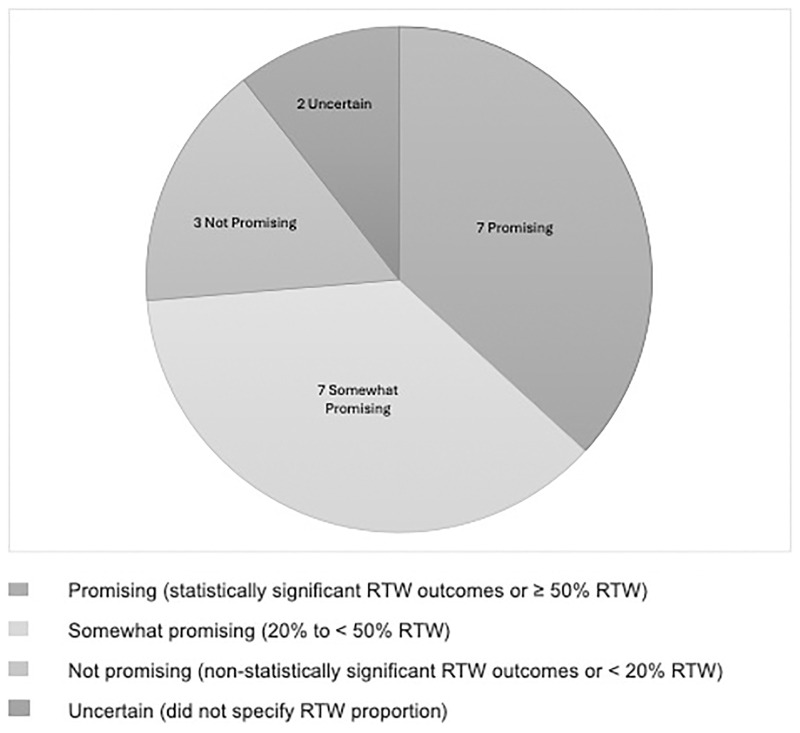
Studies categorized according to level of intervention promise.

Reliability of the screening process of titles, abstracts, and full texts was high with an average agreement percentage ranging from 77 to 84% between reviewers. The initial search of the online databases identified 4,314 potentially relevant articles. A search of the grey literature obtained 863 documents or articles potentially relevant to promoting RTW in people living with Long COVID. Once duplicates were removed, 2,579 unique studies were included for the screening of titles and abstracts. The full texts of 304 articles were screened, and from that process, 19 research articles and 12 relevant clinical practice guidelines were found. Thus, 31 relevant articles were included for data extraction. Fig 1 shows the PRISMA flow chart of our article search and relevance selection process. Fig 2 shows the number of studies included based on level of intervention promise.

### 3.1. Descriptive summaries of included studies

Nineteen relevant intervention studies were included (see S1 Table). Furthermore, a descriptive summary of the intervention studies including intervention details, results, and proportion of RTW was created (see S2 Table). A summary of key messages for RTW recommendations from 12 clinical practice guidelines (see S3 Table) was also created to synthesize findings identified in the literature [19]. Summaries were organized based on relevance to the study question.

Relevant articles included diverse study designs including three quasi-experimental studies [24–26], three randomized controlled trials (RCTs) [27–29], five cohort studies [6,30–33], 4 observational studies [34–37], two interventional studies [38,39], and two case reports [40,41]. We included eight studies from Germany, one from Austria, three from Norway, one from Spain, one from France, two from Canada, two from the USA, and one from Japan. Notably, more than half of the samples in all studies were composed of females, and the majority (84.2%) had a higher female sample.

Regarding the interventions, most lasted over three weeks (78.9%), with one study’s intervention lasting only three and a half days while the others ranged between nine and 42 days. All studies included participants over 18 years of age who met the Long COVID definition of either the World Health Organization or the National Institute for Health and Care Excellence [2,42]. Nine studies required confirmed acute COVID-19 infection [24,26–28,30,31,33,39,40], while four allowed suspected or confirmed infection [31,32,37,38], and six had vague inclusion criteria without specifics related to COVID-19 [29,34–36,38,41].

### 3.2. Key intervention findings

#### Promising or somewhat promising strategies/approaches to treatment.

The relevant studies highlighted several successful approaches to rehabilitation and treatment for managing Long COVID or RTW. These programs ranged from specialized, individualized, multiple-session rehabilitation to concentrated three-day rehabilitation programs and exploratory medical procedures.

#### Multidisciplinary rehabilitation and clinical interventions – Physical health.

Rehabilitation programs that showed promising results for RTW included respiratory therapy, muscular training, a mental health component, and educational interventions [6,24,25]. Three studies found that multidisciplinary, individualized treatment plans were recommended to address Long COVID symptoms like fatigue, autonomic dysfunction, and cognitive deficits, which impact workplace sustainability [6,25,30]. Altmann et al. (2023) found breathing exercises and respiratory treatments facilitated by physical therapists aided individuals in managing symptoms of shortness of breath and fatigue crucial for RTW [25]. In addition, they found monitoring oxygen levels during rehabilitation and daily activities helped pace and manage PEM, contributing to sustainable RTW [25]. It was found that RTW readiness from a physical perspective, due to the variability of symptoms experienced, was challenging and required input from multiple experts and specialists [6]. Garbsch et al. (2024) employed both traditional and non-traditional therapies in a multidisciplinary inpatient rehabilitation program combining active, cognitive, and passive therapies resulting in significant workability improvement at 6 months post-rehabilitation compared to admission, with no notable sex differences [30]. In addition to physical rehabilitation, relaxation treatment and psychological counselling, this intervention also included aqua fitness, inspiratory muscle training, nutrition education, heat therapy, and group exercise [30].

Three other studies with multidisciplinary Long COVID rehabilitation programs, employed more non-traditional methods [24,36,39]. Frisk et al. (2023) demonstrated that micro-choices (3-day concentrated program) can enhance physical activity and functioning beyond symptom monitoring alone [24]. By incorporating patient education, individualized exercise to break inflexible patterns, physical activity training, and brief mindfulness sessions, the intervention led to a significant reduction in sick leave [24]. Furthermore, Frisk et al. (2025) also found that the concentrated, micro-choice-based treatment for Long COVID led to sustained improvements in sick leave, fatigue, and functional status at the one-year follow-up [39]. Schmid et al. (2024) demonstrated a substantial and sustained increase in work capacity following a multimodal rehabilitation program [36]. The intervention consisted of a wide range of more non-traditional therapies, including nutrition therapy, hydrotherapy/thermotherapy, mind-body medicine, phytotherapy, naturopathy, and traditional exercise therapy, individualized to each patient’s symptoms and rehabilitation goals [36].

Muller et al. (2023) showed that rehabilitation targeting physical and neuropsychological health for individuals with Long COVID can lead to specific symptom reduction but not overall global fatigue and thus was considered somewhat promising [35]. This study measured various aspects of each symptom across two-time points with multiple tests and outcome measures, which documented a decreased prevalence in all symptoms except for fatigue [35]. Muller et al. (2023) found that despite the significant self-reported health improvements in people living with Long COVID, especially healthcare workers, they continued to suffer from poor workability with 72% of participants still unable to RTW post-rehabilitation [35,38]. In the 1-year follow-up study by Muller et al. (2024), the median WAI total score indicated poor workability that worsened significantly over time [34]. Thus, rehabilitation programs that lacked individualized treatment or had too many outcome measures were less effective and did not improve workability [35,38].

#### Rehabilitation and clinical interventions – Mental and cognitive health.

Altmann et al. (2023) also found that psychological counselling and coping strategies, such as mindfulness, helped manage anxiety and depression stemming from uncertainty about recovery, grief over lost abilities, and symptoms of Long COVID itself [25]. Early education on symptom management, pacing, graded return to activity, and energy conservation were emphasized as key components of interventions to manage Long COVID and build capacity for RTW [6,31]. Garcia-Molina et al. (2022) found that cognitive rehabilitation was effective for RTW in addressing cognitive impairments such as memory loss, brain fog, and concentration difficulties [26]. Interventions like cognitive pacing, compensatory strategies, and memory exercises enhanced mental clarity and cognitive function, supporting RTW [26]. In addition, Garcia-Molina et al. (2022) found that symptom monitoring tools like daily diaries or trackers helped individuals identify symptom patterns, enabling targeted interventions and a smoother RTW transition [26]. Nerli et al. (2024), highlighted the significance of a brief outpatient rehabilitation intervention based on the Cognitive Activation Theory of Stress in supporting RTW for people living with Long COVID [28]. By addressing cognitive expectancies that influence symptom persistence, this intervention helped modify patients’ stress response and improve their symptom management, leading to short-term and sustained RTW outcomes at the end of the program and six months follow-up.

#### Workplace strategies.

Ongoing monitoring and individualized treatment plans at the workplace were considered promising, with graduated RTW processes recommended to accommodate the fluctuating nature of Long COVID symptoms [6,31]. According to Brehon et al. (2022) and Garcia-Molina et al. (2022), RTW planning with modified, gradual returns (including adjusted hours and duties) was essential to accommodate fluctuating and episodic Long COVID symptoms and led to positive RTW outcomes [6,26]. Frisk et al. (2023) add another important component including RTW planning and suggestions for policy changes to sick leave to allow greater flexibility, remote work options, and time off for people living with Long COVID due to the episodic nature of symptoms [24]. These research studies found that graduated RTW plans with modified work responsibilities were promising for RTW planning [6,24,26]. Early intervention (less than 6 months) was linked to higher RTW success rates, as shorter times between symptom onset and treatment correlated with improved outcomes and a higher likelihood of successful RTW [6].

#### Exploratory medical procedures.

Other promising interventions included specialized rehabilitation programs for Long COVID such as using Enhanced External Counter Pulsation (EECP) [25,33]. Sathyamoorthy et al. (2022) found that EECP improved blood flow and reduced cardiac strain, helping alleviate symptoms like persistent fatigue and shortness of breath, which in turn boosted physical endurance and overall well-being, facilitating a more sustainable RTW [33]. The sample size for this study was small (n = 16), thus this approach should be considered exploratory. In addition, a pilot RCT by Uswatte et al. (2024) explored the feasibility of Constraint-Induced Cognitive Therapy (CICT) for people living with Long COVID-related cognitive impairment and IADL dysfunction, demonstrating high completion rates and participation satisfaction [29]. This 36-hour intervention incorporated speed of processing of training, structured IADL practice, and real-world skill transfer, resulting in improvements in brain fog, daily functioning, and psychological well-being [29]. The results are promising and suggest that CICT may facilitate RTW by enhancing cognitive function and independence, thus highlighting its potential as a rehabilitation treatment for Long COVID [29]. Again, the sample size of this pilot study was small (n = 14), thus CICT should be considered exploratory. Lastly, a case report demonstrated improved workability from critical to good using pulse Electromagnetic Field Therapy (EMF) by treating a patient with high magnetic flux density over 5 weeks to manage Long COVID fatigue [41]. This rehabilitation approach is exploratory and may improve RTW results by improving fatigue and overall well-being but true effects on RTW remain unknown (i.e., classified as ‘uncertain’ effects) [41].

#### Approaches deemed not promising.

Some interventions were found to have little impact on RTW in people living with Long COVID. Derksen et al. (2023) completed an intervention involving personal pilots and digital rehabilitation, which was unsuccessful in improving RTW outcomes for people living with Long COVID, despite reductions in symptoms [27]. Although personal pilots provided valuable support and helped patients navigate healthcare, their lack of therapeutic training and the reliance on asynchronous digital interventions without a physiotherapist’s supervision limited effectiveness [27]. While symptom reduction predicted higher social participation, it did not translate into improved workability, suggesting that broader social factors beyond individual symptom management influence workability [27].

Unsuccessful rehabilitation treatment options often involved aggressive or graded exercise plans without accounting for PEM, which worsened symptoms [38]. Hasenoehrl et al.’s (2023) rehabilitation intervention focused on physical conditioning for individuals with Long COVID to improve endurance, muscular strength, and workability but did not report RTW proportion leaving the intervention to have unknown RTW effects [38]. Although exercise led to significant improvements in physical fitness, psychological outcomes, and reported workability, the individual gains were more pronounced in those with severe fatigue compared to those with milder symptoms [38]. Despite these benefits, the intervention did not significantly enhance RTW overall suggesting that while exercise may lead to improvements in some outcomes, it does not address all the complex components of RTW, especially in individuals with varying levels of fatigue, PEM, and other Long COVID symptoms [38].

The study by Kupferschmitt et al. (2023) showed that a multimodal inpatient rehabilitation program utilizing psychotherapy-based approaches achieved only a marginal 2% improvement in RTW rates, indicating limited effectiveness for RTW recovery among individuals with Long COVID [37]. While patients without signs of PEM benefited from psychological and physical interventions, their psychological burden was lower than that of patients with psychosomatic or psychocardiological conditions, potentially diminishing the impact of the psychotherapy-based methods [37]. Consequently, this approach was regarded as less promising, as the non-curative nature of rehabilitation and the absence of tailored RTW-focused strategies may have contributed to its limited success in facilitating RTW reintegration [37].

#### Approaches with uncertain promise.

Two interventions were deemed of uncertain promise because they did not specifically examine RTW rates after the intervention. However, both studies reported positive outcomes on other work-related measures. Wagner et. al. studied the use of pulsed electromagnetic field therapy and found that the intervention improved self-rated workability from ‘critical’ (21.5 points) before treatment to ‘good’ (40 points) after the intervention [41]. As discussed above, Hasenoehrl et. al. studied physical exercise and reported some improvement in self-reported workability in health care workers [38]. Both interventions will need to be evaluated for their impact on actual RTW outcomes.

### 3.3. Recommendations related to RTW from published guidelines

Multiple guidelines for managing Long COVID have been published in various countries and by the World Health Organization [42,43–53]. Most of these provide recommendations for RTW, which will be summarized here.

#### Individualized support.

Guidelines for RTW recommendations emphasize tailored support acknowledging unique needs, symptoms, and personal situations as well as the importance of early intervention, integration of healthcare services, and personalized rehabilitation plans [43]. Proactive involvement of individuals from various areas (clinical, workplace, unions, insurance/compensation, etc.) is important for finding solutions for successful RTW [43]. Individualized, tailored treatment recommendations include starting aerobic exercises at lower intensities with conservative progression and careful symptom monitoring to avoid PEM [48,54]. In addition, it has been recommended that the needs of individual workers be recognized and addressed regarding challenges that may particularly impact RTW, including activity-induced mental and cognitive fatigue, sleep, mood disturbances, and persistent cognitive problems [48,54]. Adequate rest and medical assessment at four weeks post-symptom onset appear crucial for preventing Long COVID symptomology, followed by referrals to specialists and tailored rehabilitation programs as indicated [43]. Treatment should focus on Long COVID symptoms that impact work readiness, with ongoing follow-up, symptom monitoring, and providing self-management resources and contact information for flare-ups [49,52]. Lastly, individualized accessibility recommendations ensure that specific therapies such as pulmonary rehabilitation are accessible to equity-deserving populations, use clear language for ease of interpretability, overcome transportation needs or provide virtual options to support participation in treatment [54].

#### Workplace accommodations.

Workplace accommodations are crucial for people with mild to moderate Long COVID symptoms to stay engaged and safely RTW [49,53]. Recommendations for common accommodations for fatigue include reduced work hours, flexible scheduling, and avoidance of tasks with high cognitive demands [49,53]. Gradual RTW is often necessary due to the unpredictable nature of Long COVID symptoms [44,46,49]. Work task or work environment modifications, guided by healthcare provider-directed restrictions and limitations, can often enable a safe and timely RTW [45,50,53]. Physician and/or clinician guidance is essential to ensure that accommodations preserve functionality and protect against the negative health impacts of worklessness [50,53]. RTW planning meetings have been recommended before gradual RTW to address needs, review workloads, involve union and/or employer representatives and explore modifiable duties and responsibilities [45,48,49]. Some modifications recommended include start and end time flexibility, modifiable hours/days, workload changes, increased frequency of breaks, microbreaks, modified or alternative duties, support mechanisms (i.e. a buddy or work from home options), and equipment adjustments [44–46,49]. Other recommendations include modifying policies (i.e. sick leave, bullying/harassment, confidentiality around sharing positive testing, etc.), fostering autonomy over scheduling (i.e. remote work, self-management of symptoms, etc.) and workplace goal setting (i.e. modifying targets) [49].

A key workplace accommodation recommendation for people living with Long COVID involves reviewing and changing policies that address disability benefits and employment protection pathways [49,51]. Actions should be taken to adjust policies concerning employment rights and sick pay as well as provide flexibility in leave due to the episodic nature of Long COVID [49,51]. It has been recommended that all countries review and update their wage replacement benefit packages and access to disability benefits for Long COVID [51].

#### Managers play a critical role.

Managers play a significant role in facilitating a successful RTW as they are often the first point of contact for workers considering RTW after Long COVID [44,46,49,50]. RTW for people living with Long COVID requires support and collaboration among multiple parties, including the worker, employer, line manager, and healthcare professionals [43,44,46]. Employers, Human Resources, and supervisors play key roles in providing flexible support, and implementing job modifications and work adjustments from gradual RTW plans to support recovery in the workplace [43,44,46]. Supportive attitudes, active listening, and an accommodating nature are vital for RTW support [44,46]. In addition, recommendations for managers include staying in touch during absences from work, preparing for the worker’s return, conducting an RTW discussion, providing support during early RTW days, and implementing modifications [44,46]. Ongoing discussions and regular reviews are essential to address the episodic nature of Long COVID to ensure sustained workability [43,50]. The main objective of discussions should be a gradual and adaptable RTW, alongside support for sustainable approaches to RTW [43,49]. Sustainable employment is beneficial for health from the broader disability perspective as we need work and/or productivity for our overall health and functioning as well as for the global economy [43,49].

#### Clinical intervention or rehabilitation programs.

For individuals with significant health concerns, such as chest pain or cognitive dysfunction, it is recommended to carry out specific assessments and clearances before resuming strenuous or safety-critical work [43,50]. Physical exercise as an intervention is contraindicated for those experiencing PEM and a fit note should be obtained before starting the program [43,50]. Strengthening exercises should be introduced gradually, and educational modules should be adapted for Long COVID specific challenges and be introduced early [54]. Occupational therapists and psychologists are recommended for Long COVID treatment, in particular for assessing and monitoring RTW readiness [54]. Interventions should always incorporate RTW-specific items in outcome measures as part of the assessment and follow-up to determine if the intervention was successful [50,54].

An important recommendation from clinical practice guidelines related to RTW interventions is to use a holistic, multidisciplinary approach to assess how lives, especially work lives, have been impacted by Long COVID [52]. RTW discussions in programs should involve occupational health services and health professionals to support recovery [43,51,52]. Shared decision-making should guide the discussion with agreement on support and rehabilitation strategies tailored to an individual’s needs [50,52]. Creating individualized rehabilitation programs that address all problem areas, including RTW, is crucial [52]. It is recommended that interventions should be tailored to address sleep hygiene, mental health concerns such as depression, stress, and anxiety, assessment and treatment of occupational demands, and supervised physical exercise [48]. Effective RTW planning should focus on overcoming obstacles using a biopsychosocial approach and maintaining adaptability to support people living with Long COVID throughout their recovery [43,49]. This includes setting realistic expectations, focusing on what can be achieved, and addressing both physical and psychological functioning [43]. At this time, guidelines state that initial examinations or assessments prior to interventions should include the one-minute sit-to-stand test and three-minute active stand test to screen for orthostatic intolerance and PEM [48].

## 4. Discussion

Findings of this scoping review indicate that multidisciplinary Long COVID rehabilitation programs, employing traditional and non-traditional methods, were promising [6,24–26,36,39]. Key strategies for treatment highlight effective RTW interventions combining physical and mental health support, including respiratory and muscular training, psychological counselling, cognitive rehabilitation, and symptom management tools [6,24–26]. Other promising exploratory approaches to Long COVID treatment and RTW included Constraint-Induced Cognitive Therapy (CICT), and Enhanced External Counter Pulsation (EECP) [29,33,41]. Electromagnetic Field Therapy (EMF), approach demonstrated improved workability; however true effects on RTW remain unknown [41].

The findings of this review underscore several critical insights into the current state of RTW interventions for individuals with Long COVID. First, there is a notable scarcity of rigorous studies specifically evaluating the effectiveness of RTW interventions tailored for Long COVID. This highlights the research gap which limits the evidence base needed to guide clinicians and policymakers in supporting individuals with Long COVID effectively. Secondly, valuable insights can be obtained from existing clinical practice guidelines, which offer a range of RTW recommendations. These guidelines, although not always validated specifically for Long COVID patient population, provide practical suggestions that can be applied and adapted to this emerging area of practice. The third key takeaway is the complexity of RTW for individuals with Long COVID, as the episodic and unpredictable nature of the condition presents unique challenges. Achieving successful RTW for these individuals typically demands sustained, adaptable support. This includes individualized, targeted healthcare and rehabilitation interventions to manage symptoms, workplace accommodations to address fluctuating abilities and comprehensive social support systems. Workplace accommodations such as flexible hours and gradual RTW are recommended to address fluctuating symptoms of Long COVID and align with broader RTW recommendations for work disability caused by health conditions or illness [6,42,51,52]. Interventions with any success for RTW included RTW planning with modified duties and suggested policy updates for flexible sick leave.

Studies classified as ‘uncertain’ included return-to-work (RTW) outcomes but did not report the specific proportion of participants who returned to work. Given this uncertainty regarding RTW success rates, these studies were not included in forming conclusions or recommendations related to ‘promising’ interventions. We recommend that further research is needed on these interventions. While some interventions for Long COVID, such as constraint-induced cognitive therapy or enhanced external counter pulsation, have been classified as ‘promising,’ their real-world implementation may be challenging due to feasibility factors. These interventions often require substantial financial resources, specialized equipment, or advanced clinician training, which may limit their accessibility across diverse healthcare settings. As Long COVID care continues to evolve, future research and policy initiatives must address issues of scalability, cost-effectiveness, and equitable access to ensure broader implementation.

RTW recommendations from international guidelines emphasize individualized, multidisciplinary support involving clinicians, employers, and policy updates to facilitate a sustainable RTW process [42,44–46,49]. Key findings highlight employer support from supervisors or managers, gradual RTW, clinician or physician oversight of RTW plans, and changes to policies/procedures for flexibility to account for the episodic nature of Long COVID [43,44,55,46,50,53]. The significant challenges for RTW with Long COVID must be acknowledged; indeed, these challenges are currently recognized as symptomatic of Long COVID, since most individuals with Long COVID struggle to RTW in any capacity [52].

### Interventions that should be avoided

#### Resistance training or aggressive exercise.

Aggressive exercise such as resistance exercise training or graded activity, is often counterproductive for individuals with Long COVID due to the high prevalence of PEM and the episodic nature of fluctuating symptoms like fatigue, autonomic dysfunction, and cognitive impairments [2,4,13,16,38]. Unlike traditional rehabilitation where exercise is used to improve functional abilities and work readiness, for individuals with Long COVID experiencing PEM, excessive physical exertion can exacerbate symptoms leading to prolonged recovery setbacks [2,13,16]. Graded exercise for those with PEM is currently contraindicated and studies indicate that interventions ignoring PEM risk worsening fatigue and other symptoms, thus reducing the effectiveness of rehabilitation and potentially delaying RTW [2,13,16]. Therefore, baseline measurements and monitoring symptoms must be a component of treatment where physical conditioning is involved [31]. Overall, the current recommendation is a conservative, individualized approach to physical activity that prioritizes pacing and symptom monitoring to ensure safety and RTW support for individuals living with Long COVID.

#### Digital or unsupervised treatment.

Unsupervised or self-directed rehabilitation programs via an app for Long COVID recovery may be insufficient, as they often lack the personalized oversight necessary to manage complex, fluctuating symptoms effectively. Less effective treatment approaches, such as self-directed via app programs, often overlooked crucial elements such as pacing, orthostatic intolerance and PEM hindering RTW and/or led to no changes in global fatigue [27]. For those with symptoms such as fatigue, brain fog, and autonomic dysfunction, supervision and modifications are frequently needed, which digital platforms alone often cannot support [2,27,38,42]. As such, programs that do not provide proper training for people living with Long COVID or rely on digital interventions without supervision were found to be less effective [27]. These treatment programs also have the potential to worsen PEM and impact overall recovery for individuals with Long COVID, potentially delaying RTW [27,38]. Without direct feedback from clinicians, patients may overexert themselves or miss subtle warning signs of PEM, risking flare-up hindering overall recovery and delaying RTW [13,27]. This indicates the need for a more comprehensive approach that is supervised, multidisciplinary, and individualized, targeting the individual’s personal and contextual needs. Virtual rehabilitation treatment with mindfulness, and physical and sensory exercises but no clinician supervision was found to lead to symptom reduction but no RTW, highlighting the need for clinician support as stated in other sources [6,27,38]. Studies show that in-person, supervised care from clinicians is vital to ensure effective and safe pacing, modifications to programs, and comprehensive support necessary for sustainable RTW [27,56].

#### Excessive outcome measurements.

Although Long COVID rehabilitation can address physical and psychological symptoms, programs addressing every symptom with multiple outcome measures may fall short in RTW preparation and managing fatigue [35]. Using too many outcome measures or maximal testing with a performance-based assessment of workability may overwhelm patients and clinicians, detracting from effective rehabilitation. Due to a lack of treatment options for the over 200 possible symptoms of Long COVID, clinicians may feel pressured to document all problem areas. With too many measures, the attention on meaningful progress or change can be diluted, making it difficult to identify which interventions truly support Long COVID recovery for RTW. Individuals with Long COVID often struggle with global fatigue and PEM, which may add cognitive and physical strain, further complicating the recovery process [13,16]. They often require pacing and energy conservation strategies to manage symptoms; thus, intensive programs with multiple outcome measures can be additionally fatiguing, exacerbating PEM or worsening symptoms for these individuals [31]. There is a need to streamline outcome metrics to focus on core areas such as functional capacity, symptom management, and RTW readiness to allow clarity for more actionable insights, helping both clients and clinicians with targeted support that aligns with the unique and variable needs of each person with Long COVID.

### Limitations

We acknowledge some limitations with this scoping review, including the potential for publication bias that may skew findings toward positive outcomes. We also did not conduct a quality appraisal of included studies, thus there is potential for bias within the included studies. Additionally, the diversity of interventions and varying study designs across the included research could impact the consistency and comparability of results. Limitations in the data sources, such as inclusion of studies with small sample sizes, a lack of longitudinal follow-up, or inclusion of people with a large spectrum of symptoms may also affect generalizability of findings. This study included mainly English-language studies, which may have missed some important literature published in other languages. We included a very broad range of interventions, which included some exploratory medical interventions such as EECP. While interesting, these were not comparable to other commonly used RTW interventions and should be evaluated and assessed separately. Furthermore, given the nature of Long COVID and its evolving research means that some relevant interventions may be inadequately represented in the literature. The dynamic and rapidly developing nature of this literature will require updates. Lastly, the generalizability of these findings to other healthcare contexts should be approached with caution, as variations in healthcare systems, populations, and resources may influence the applicability of the results.

### Recommendations for future research

Despite current rehabilitation recommendations and research studies for Long COVID, further research is necessary to refine rehabilitation programs to improve workability and facilitate successful RTW [6,24,31]. Further research is necessary to determine whether Long COVID is a single condition or a group of conditions requiring distinct treatment and monitoring [3]. This can inform which professionals are necessary for multidisciplinary or transdisciplinary teams [20,25]. It is inevitable that Long COVID, like other chronic conditions, will require ongoing management due to its multidimensional, episodic, and fluctuating nature [13]. Thus, research to determine similarities and differences between Long COVID trajectories and other chronic conditions may help inform Long COVID treatment approaches [3,26]. In Canada, individuals with Long COVID often face a minimum 3-month waiting period before accessing treatment such as rehabilitation programs, which may be a barrier to RTW [16]. Thus, research on whether earlier education or access to rehabilitation interventions benefits individuals with lingering COVID-19, but not yet diagnosed with Long COVID, is necessary [16].

Future research should explore adapting rehabilitation programs for chronic illnesses for those living with Long COVID to improve workability outcomes [6,35]. Much like other chronic illnesses, early rehabilitation for RTW needs to be explored for individuals with Long COVID as research findings correlate with RTW improvements for those with less time between symptom onset and rehabilitation [6]. It has been highlighted that flexible work arrangements improve RTW for individuals with Long COVID thus, this should be incorporated into rehabilitation programs and tested in future studies [6]. Additionally, personalized rehabilitation approaches incorporating work simulation of specific job demands and managing the severity of fatigue should be explored with education on pacing included [31]. The benefits of early education on pacing, energy conservation, and compensatory cognitive strategies need to be tested in future research studies as individuals with Long COVID and current research point towards potential success for RTW in these areas [6,26]. Research on indicators for successful RTW and prognostic factors is also needed.

Exploring discrepancies across Long COVID rehabilitation programs and approaches is also necessary to determine their true correlations with RTW success. Specifically, the requirement for medical clearance or fit notes varies by employer, with some programs deeming RTW as a sign of symptom recovery/improvement while others view it as part of an ongoing RTW journey [6,31,33]. Furthermore, variations in communication approaches regarding Long COVID affect the certainty of findings. Some programs prioritize autonomy and discretion, while others encourage openness about COVID-19 symptoms and infection. This is vital in the broader scope of Long COVID rehabilitation where agreement in diagnosis, definition, and prognosis across clinical team members is necessary [2]. Understanding these discrepancies in current rehabilitation approaches can lead to the standardization of best practices and improve rehabilitation outcomes for RTW in Long COVID [27,31].

Despite the availability of numerous rehabilitative approaches to RTW for Long COVID, specific rehabilitative treatment modalities and approaches for Long COVID rehabilitation are needed. To date, most approaches have considered a trial-and-error approach by applying strategies used with other populations, using traditional rehabilitation strategies, and/or using treatments that target individual symptoms; however, specific Long COVID treatment is necessary [27,31]. Longitudinal studies are crucial in understanding the sustainability of rehabilitation for RTW alongside ongoing aftercare support due to the episodic nature and fluctuations of Long COVID [6]. Most programs have incorporated a mental health component, but further research is necessary to explore the psychological aspects of work readiness and to develop strategies to enhance mental resilience and optimize RTW outcomes for individuals with Long COVID [24,25]. Health systems and policy research is also needed on the effect of disability benefits (short-term or long-term) on RTW.

## 5. Conclusion

Our findings highlight the importance of targeted, individualized rehabilitation approaches to support recovery and facilitate sustainable RTW for people living with Long COVID. There is a scarcity of rehabilitation tools or protocols specifically designed to address Long COVID symptomology. This review highlights key elements of promising interventions such as integrating physical and mental health support, tailored and supervised pacing, and flexible workplace accommodations that align with the fluctuating nature of Long COVID symptoms. While some existing strategies have shown promise, other interventions do not appear to provide adequate support to promote RTW. Findings of this review highlight the necessity of refining rehabilitation programs to provide multidimensional, patient-centered, safe, and effective approaches to promoting RTW for this population. There is an urgent need to develop effective programs for individuals with Long COVID to promote recovery and RTW, especially given the social and economic impact of this condition and the current lack of appropriate RTW supports.

## Supporting information

S1 Text Supporting information file. (DOCX)

S1 Table Descriptive characteristics of intervention studies. (DOCX)

S2 Table Categories and key messages of interventions studies for long COVID. (DOCX)

S3 Table Recommendations for return-to-work from guidelines. (DOCX)

## References

[1] Merad M, Blish CA, Sallusto F, Iwasaki A. The immunology and immunopathology of COVID-19. Science. 2022;375(6585):1122–7. doi: 10.1126/science.abm8108 35271343 PMC12828912

[2] Office of the Chief Science Advisor of Canada Task Force on Post-COVID-19 Condition in Canada: What we know, what we don’t know, and a framework for action. 2022 https://science.gc.ca/site/science/en/office-chief-science-advisor/initiatives-covid-19/post-covid-19-condition-canada-what-we-know-what-we-dont-know-and-framework-action. Accessed October 8, 2025

[3] Dennis A, Wamil M, Alberts J, Oben J, Cuthbertson DJ, Wootton D, et al. Multiorgan impairment in low-risk individuals with post-COVID-19 syndrome: a prospective, community-based study. BMJ Open. 2021;11(3):e048391. doi: 10.1136/bmjopen-2020-048391 33785495 PMC8727683

[4] Tabacof L, Tosto-Mancuso J, Wood J, Cortes M, Kontorovich A, McCarthy D, et al. Post-acute COVID-19 Syndrome Negatively Impacts Physical Function, Cognitive Function, Health-Related Quality of Life, and Participation. Am J Phys Med Rehabil. 2022;101(1):48–52. doi: 10.1097/PHM.0000000000001910 34686631 PMC8667685

[5] Inclusively. The Immense Impact of Long COVID on Workers (And What Employers Can Do About It). December 2022. https://www.inclusively.com/immense-impact-of-long-covid-on-workers/. Accessed October 8, 2025

[6] Brehon K, Niemeläinen R, Hall M, Bostick GP, Brown CA, Wieler M, et al. Return-to-Work Following Occupational Rehabilitation for Long COVID: Descriptive Cohort Study. JMIR Rehabil Assist Technol. 2022;9(3):e39883. doi: 10.2196/39883 36094442 PMC9484483

[7] Brehon K, Miciak M, Hung P, Chen SP, Perreault K, Hudon A, et al. “None of us are lying”: an interpretive description of the search for legitimacy and the journey to access quality health services by individuals living with Long COVID. BMC Health Serv Res. 2023;23(1):1396. 38087299 10.1186/s12913-023-10288-y PMC10714615

[8] Anderson E, Hunt K, Wild C, Nettleton S, Ziebland S, MacLean A. Episodic disability and adjustments for work: the ‘rehabilitative work’ of returning to employment with Long Covid. Disability & Society. 2024;40(5):1239–61. doi: 10.1080/09687599.2024.2331722

[9] Canada Go. COVID-19 longer term symptoms among Canadian adults: Fourth report. 2024. https://www.canada.ca/en/public-health/services/diseases/coronavirus-disease-covid-19/long-term-symptoms-among-canadian-adults-fourth-report.pdf

[10] Quinn KL, Lam GY, Walsh JF, Bhéreur A, Brown AD, Chow CW, et al. Cardiovascular Considerations in the Management of People With Suspected Long COVID. Can J Cardiol. 2023;39(6):741–53. doi: 10.1016/j.cjca.2023.04.003 37030518 PMC10160565

[11] Choutka J, Jansari V, Hornig M, Iwasaki A. Unexplained post-acute infection syndromes. Nat Med. 2022;28(5):911–23. doi: 10.1038/s41591-022-01810-6 35585196

[12] Davis HE, McCorkell L, Vogel JM, Topol EJ. Long COVID: major findings, mechanisms and recommendations. Nat Rev Microbiol. 2023;21(3):133–46. doi: 10.1038/s41579-022-00846-2 36639608 PMC9839201

[13] Vink M, Vink-Niese A. The updated NICE guidance exposed the serious flaws in CBT and graded exercise therapy trials for ME/CFS. Healthcare. 2022;10(5). 10.3390/healthcare10050898 PMC9141828 35628033

[14] Müller K, Zwingmann K, Auerswald T, Berger I, Thomas A, Schultz A-L, et al. Rehabilitation and Return-to-Work of Patients Acquiring COVID-19 in the Workplace: A Study Protocol for an Observational Cohort Study. Front Rehabil Sci. 2022;2:754468. doi: 10.3389/fresc.2021.754468 36188830 PMC9397694

[15] Pouliopoulou DV, Macdermid JC, Saunders E, Peters S, Brunton L, Miller E, et al. Rehabilitation Interventions for Physical Capacity and Quality of Life in Adults With Post-COVID-19 Condition: A Systematic Review and Meta-Analysis. JAMA Netw Open. 2023;6(9):e2333838. doi: 10.1001/jamanetworkopen.2023.33838 37725376 PMC10509723

[16] Thille P, Cooper JE, Jansson A, Leclair L, Parsons J, Webber S, et al. Advocating for Long COVID Rehabilitation Support in Manitoba: An Environmental Scan.: Manitoba Academic Rehabilitation Sciences Covid Interest Group (MARSCI); 2022.

[17] von Zweck C, Naidoo D, Govender P, Ledgerd R. Current Practice in Occupational Therapy for COVID-19 and Post-COVID-19 Conditions. Occup Ther Int. 2023;2023:5886581. doi: 10.1155/2023/5886581 37250066 PMC10219768

[18] Manhas KP, O’Connell P, Krysa J, Henderson I, Ho C, Papathanassoglou E. Development of a Novel Care Rehabilitation Pathway for Post-COVID Conditions (Long COVID) in a Provincial Health System in Alberta, Canada. Phys Ther. 2022;102(9):pzac090. doi: 10.1093/ptj/pzac090 35778936 PMC9384405

[19] Levac D, Colquhoun H, O’Brien KK. Scoping studies: advancing the methodology. Implement Sci. 2010;5:69. doi: 10.1186/1748-5908-5-69 20854677 PMC2954944

[20] DeMars J, Durand-Moreau Q, Branton E, Nowrouzi-Kia B, Gross DP, participants of the 2024 Canadian Symposium on Long COVID Return to Work Session. Improving Occupational Rehabilitation for People Living with Long COVID. J Occup Rehabil. 2025;35(1):1–3. doi: 10.1007/s10926-024-10267-y 39878862

[21] Nagra G, Hung P, Peters MR, Guptill C, Ezeugwu VE, Cooper L, et al. Considerations for engaging in patient-oriented research with injured workers. Front Health Serv. 2025;5:1589643. doi: 10.3389/frhs.2025.1589643 40535202 PMC12174379

[22] Gardner B, Smith L, Lorencatto F, Hamer M, Biddle SJH. How to reduce sitting time? A review of behaviour change strategies used in sedentary behaviour reduction interventions among adults. Health Psychol Rev. 2016;10(1):89–112. doi: 10.1080/17437199.2015.1082146 26315814 PMC4743603

[23] Moore SA, Hrisos N, Flynn D, Errington L, Price C, Avery L. How should long-term free-living physical activity be targeted after stroke? A systematic review and narrative synthesis. Int J Behav Nutr Phys Act. 2018;15(1):100. doi: 10.1186/s12966-018-0730-0 30333027 PMC6192196

[24] Frisk B, Jürgensen M, Espehaug B, Njøten KL, Søfteland E, Aarli BB, et al. A safe and effective micro-choice based rehabilitation for patients with long COVID: results from a quasi-experimental study. Sci Rep. 2023;13(1):9423. doi: 10.1038/s41598-023-35991-y 37296140 PMC10252160

[25] Altmann CH, Zvonova E, Richter L, Schüller PO. Pulmonary recovery directly after COVID-19 and in Long-COVID. Respir Physiol Neurobiol. 2023;315:104112. doi: 10.1016/j.resp.2023.104112 37406842

[26] Garcia-Molina A, Garcia-Carmona S, Espina-Bou M, Rodriguez-Rajo P, Sanchez-Carrion R, Ensenat-Cantallops A. Neuropsychological rehabilitation for post-COVID-19 syndrome: Results of a clinical program and six-month follow up. Neurologia. 2022. 10.1016/j.nrleng.2022.06.007 PMC9476330 36116770

[27] Derksen C, Rinn R, Gao L, Dahmen A, Cordes C, Kolb C, et al. Longitudinal Evaluation of an Integrated Post-COVID-19/Long COVID Management Program Consisting of Digital Interventions and Personal Support: Randomized Controlled Trial. J Med Internet Res. 2023;25:e49342. doi: 10.2196/49342 37792437 PMC10563866

[28] Nerli TF, Selvakumar J, Cvejic E, Heier I, Pedersen M, Johnsen TL, et al. Brief Outpatient Rehabilitation Program for Post-COVID-19 Condition: A Randomized Clinical Trial. JAMA Netw Open. 2024;7(12):e2450744. doi: 10.1001/jamanetworkopen.2024.50744 39699896 PMC11659907

[29] Uswatte G, Taub E, Ball K, Mitchell BS, Blake JA, McKay S, et al. Long COVID Brain Fog Treatment: Findings from a Pilot Randomized Controlled Trial of Constraint-Induced Cognitive Therapy. medRxiv. 2024. 10.1037/rep0000626 PMC12323405 40310209

[30] Garbsch R, Schäfer H, Kotewitsch M, Mooren JM, Waranski M, Teschler M, et al. Sex-specific differences of cardiopulmonary fitness and pulmonary function in exercise-based rehabilitation of patients with long-term post-COVID-19 syndrome. BMC Med. 2024;22(1):446. doi: 10.1186/s12916-024-03658-8 39379918 PMC11463035

[31] Ghali A, Lacombe V, Ravaiau C, Delattre E, Ghali M, Urbanski G, et al. The relevance of pacing strategies in managing symptoms of post-COVID-19 syndrome. J Transl Med. 2023;21(1):375. doi: 10.1186/s12967-023-04229-w 37291581 PMC10248991

[32] Tanguay P, Gaboury I, Daigle F, Bhéreur A, Dubois O, Lagueux É, et al. Post-exertional malaise may persist in Long COVID despite learning STOP-REST-PACE. Fatigue: Biomedicine, Health & Behavior. 2023;11(2–4):113–28. doi: 10.1080/21641846.2023.2222199

[33] Sathyamoorthy M, Verduzco-Gutierrez M, Varanasi S, Ward R, Spertus J, Shah S. Enhanced external counterpulsation for management of symptoms associated with long COVID. Am Heart J Plus. 2022;13:100105. doi: 10.1016/j.ahjo.2022.100105 38560070 PMC10978164

[34] Müller K, Poppele I, Ottiger M, Wastlhuber A, Weber R-C, Stegbauer M, et al. Long-term course and factors influencing work ability and return to work in post-COVID patients 12 months after inpatient rehabilitation. J Occup Med Toxicol. 2024;19(1):43. doi: 10.1186/s12995-024-00443-4 39487519 PMC11529184

[35] Müller K, Poppele I, Ottiger M, Zwingmann K, Berger I, Thomas A, et al. Impact of Rehabilitation on Physical and Neuropsychological Health of Patients Who Acquired COVID-19 in the Workplace. Int J Environ Res Public Health. 2023;20(2):1468. doi: 10.3390/ijerph20021468 36674222 PMC9864141

[36] Schmid S, Uecker C, Fröhlich A, Langhorst J. Effects of an integrative multimodal inpatient program on fatigue and work ability in patients with Post-COVID Syndrome-a prospective observational study. Eur Arch Psychiatry Clin Neurosci. 2024;274(8):1983–91. doi: 10.1007/s00406-024-01792-1 38578435

[37] Kupferschmitt A, Langheim E, Tüter H, Etzrodt F, Loew TH, Köllner V. First results from post-COVID inpatient rehabilitation. Front Rehabil Sci. 2023;3:1093871. doi: 10.3389/fresc.2022.1093871 36756465 PMC9899863

[38] Hasenoehrl T, Palma S, Huber DF-X, Kastl S, Steiner M, Jordakieva G, et al. Post-COVID: effects of physical exercise on functional status and work ability in health care personnel. Disabil Rehabil. 2023;45(18):2872–8. doi: 10.1080/09638288.2022.2111467 35980383

[39] Frisk B, Jürgensen M, Espehaug B, Søfteland E, Kvale G. Sustained improvements in sick leave, fatigue and functional status following a concentrated micro-choice based treatment for patients with long COVID: A 1 year prospective uncontrolled study. J Psychosom Res. 2025;189:112023. doi: 10.1016/j.jpsychores.2024.112023 39721309

[40] Oka T. A patient who recovered from post-COVID myalgic encephalomyelitis/chronic fatigue syndrome: a case report. Biopsychosoc Med. 2023;17(1):8. doi: 10.1186/s13030-022-00260-3 36855180 PMC9971667

[41] Wagner B, Steiner M, Markovic L, Crevenna R. Successful application of pulsed electromagnetic fields in a patient with post-COVID-19 fatigue: a case report. Wien Med Wochenschr. 2022;172(9-10):227–32. 35006516 10.1007/s10354-021-00901-2 PMC8743351

[42] National Institute for Health and Care Excellence (NICE), COVID-19 rapid guideline: managing the long-term effects of COVID-19. London: NICE; 2024 Jan 25. . https://www.nice.org.uk/guidance/ng188, Accessed October 8, 2025 33555768

[43] Returning safely to work after long COVID. British Journal of Healthcare Assistants. 2022;16(1):50–50. doi: 10.12968/bjha.2022.16.1.50

[44] Burton K, Caine A, Macniven L, Porter S, Rayner C, Yarker J. COVID-19 return to work guide: for managers. Society of Occupational Medicine. 2021.

[45] The Society of Occupational Medicine. COVID-19 return to work guide For recovering workers. 2021. https://www.som.org.uk/COVID-19_return_to_work_guide_for_recovering_workers.pdf. Accessed October 8, 2025

[46] Madan I, Briggs T, Chew-Graham C. Supporting patients with long COVID return to work. Br J Gen Pract. 2021;71(712):508–9. doi: 10.3399/bjgp21X717533 34711562 PMC8544157

[47] Baril R, Clarke J, Friesen M, Stock S, Cole D, Work-Ready Group. Management of return-to-work programs for workers with musculoskeletal disorders: a qualitative study in three Canadian provinces. Soc Sci Med. 2003;57(11):2101–14. doi: 10.1016/s0277-9536(03)00131-x 14512241

[48] Lunt J, Hemming S, Elander J, Baraniak A, Burton K, Ellington D. Experiences of workers with post-COVID-19 symptoms can signpost suitable workplace accommodations. IJWHM. 2022;15(3):359–74. doi: 10.1108/ijwhm-03-2021-0075

[49] Macdonald EL, Raynor C, Yarker J. COVID -19 infection and long COVID – guide for managers. European Agency for Health and Safety at Work EUOSHA; 2021.

[50] Nurek M, Rayner C, Freyer A, Taylor S, Järte L, MacDermott N, et al. Recommendations for the recognition, diagnosis, and management of long COVID: a Delphi study. Br J Gen Pract. 2021;71(712):e815–25. doi: 10.3399/BJGP.2021.0265 34607799 PMC8510689

[51] Rajan S, Khunti K, Alwan N, Steves C, MacDermott N, Morsella A, et al. European Observatory Policy Briefs. In the wake of the pandemic: Preparing for Long COVID. Copenhagen (Denmark): European Observatory on Health Systems and Policies © World Health Organization 2021 (acting as the host organization for, and secretariat of, the European Observatory on Health Systems and Policies). 2021. 33877759

[52] Shah W, Hillman T, Playford ED, Hishmeh L. Managing the long term effects of covid-19: summary of NICE, SIGN, and RCGP rapid guideline. BMJ. 2021;372:n136. doi: 10.1136/bmj.n136 33483331

[53] Stewart-Patterson C, Bourgeois R, Martin DW. The Importance of Keeping Patients with Post-Acute Sequelae of SARS-CoV-2 Infection (Long COVID) Engaged in Work. Am Fam Physician. 2021;103(12):710. 34128623

[54] Beauchamp MK, Janaudis-Ferreira T, Wald J, Aceron R, Bhutani M, Bourbeau J, et al. Canadian Thoracic Society position statement on rehabilitation for COVID-19 and implications for pulmonary rehabilitation. Canadian Journal of Respiratory, Critical Care, and Sleep Medicine. 2021;6(1):9–13. doi: 10.1080/24745332.2021.1992939

[55] Wong J, Kudla A, Pham T, Ezeife N, Crown D, Capraro P, et al. Lessons Learned by Rehabilitation Counselors and Physicians in Services to COVID-19 Long-Haulers: A Qualitative Study. Rehabilitation Counseling Bulletin. 2021;66(1):25–35. doi: 10.1177/00343552211060014

[56] Moulaei K, Sheikhtaheri A, Fatehi F, Shanbehzadeh M, Bahaadinbeigy K. Patients’ perspectives and preferences toward telemedicine versus in-person visits: a mixed-methods study on 1226 patients. BMC Med Inform Decis Mak. 2023;23(1):261. doi: 10.1186/s12911-023-02348-4 37968639 PMC10647122

