# Supplementary material for: Return-to-work for people living with long COVID: A scoping review of interventions and recommendations
Source: PLoS One. 2025 Oct 15;20(10):e0321891. doi: 10.1371/journal.pone.0321891 (PMC12527184; doi:10.1371/journal.pone.0321891)
Supplement: S1 Text — (DOCX) [file pone.0321891.s001.docx]

**SUPPLEMENTAL APPENDIX – Search Strategy**

**Databases:** MEDLINE, EMBASE, PsycINFO, CINAHL, Scopus and Cochrane Library

Conference abstracts: Yes

Languages: All

Should we remove case reports? No

Should any other study designs be removed? No

**Description of Search Strategy**

Scoping Review: A comprehensive synthesis of evidence on the impact of rehabilitation on

work ability and return to work in individuals living with long COVID is currently lacking.

Given the emerging nature of research in this area, a full systematic review is unlikely to be

feasible due to limited available studies. Therefore, a rapid scoping review was conducted to map the scientific and grey literature on this topic, including government reports, WHO living

documents, working papers, conference proceedings, and unpublished reports. The scoping

review explored the breadth and depth of the literature, summarized available evidence, and identified knowledge gaps that may inform future research. The review adhered to the

guidelines of the Preferred Reporting Items for Systematic reviews and Meta-Analyses extension and involved a comprehensive search of multiple databases, including **MEDLINE, EMBASE, PsycINFO, CINAHL, Scopus and Cochrane Library**. We collaborated with a health science librarian (LD) at the University of Alberta to develop and conduct the search strategy.

Inclusion criteria followed the PI(E)COS framework, encompassing participants who were in

the workforce prior to contracting COVID-19, occupational rehabilitation or work

accommodation programs, relevant outcomes, and various study designs, including randomized

controlled trials, quasi-experimental studies, and observational studies. We also included clinical practice guideliens, theoretical, program development, or descriptive studies. Two independent reviewers conducted screening and data extraction, with disagreements resolved through discussion or consultation with a third reviewer.

**Example of article;**

[**https://pubmed-ncbi-nlm-nih-gov.login.ezproxy.library.ualberta.ca/36094442/**](https://pubmed-ncbi-nlm-nih-gov.login.ezproxy.library.ualberta.ca/36094442/)

**Ovid account: longcovidwork / longcovidwork**

Ovid MEDLINE(R) ALL <1946 to January 02, 2025>

Date searched: Jan 3, 2025

Results: 790

[**https://login.ezproxy.library.ualberta.ca/login?url=http://ovidsp.ovid.com/ovidweb.cgi?T=JS&NEWS=N&PAGE=main&SHAREDSEARCHID=4S9e2GQpvha92aWToyIxWOnfgj1FYWFVoM252RLEt8qC1Cb4Ca4XstOfR50ChnMmn**](https://login.ezproxy.library.ualberta.ca/login?url=http://ovidsp.ovid.com/ovidweb.cgi?T=JS&NEWS=N&PAGE=main&SHAREDSEARCHID=4S9e2GQpvha92aWToyIxWOnfgj1FYWFVoM252RLEt8qC1Cb4Ca4XstOfR50ChnMmn)

1 long COVID/ 4044

2 ((long or (long term not long term care) or long haul* or longterm or longhaul or post acute or postacute or after acute or sequela* or protracted or post-infect* or post-viral or post-discharg* or non-recover* or nonrecover* or PASC or chronic or persist* or linger* or continuing or continual) adj5 (COVID or COVID-19 or COVID19 or coronavirus* or corona virus* or 2019-nCoV or 19nCoV or 2019nCoV or nCoV or n-CoV or SARS-CoV-2 or SARS-CoV2 or SARSCoV-2 or SARSCoV2 or 2019-novel CoV or Sars-coronavirus2 or novel CoV)).mp. 21435

3 ((long or long term or long haul* or longterm or longhaul or ongoing or chronic or lengthy or protracted or persist* or linger* or continuing or continual* or post-acute or postacute or post-viral or post-discharg* or post-infect* or residual) adj2 (outcomes or symptom* or morbidity or manifestation* or issues or effects or difficulties or challeng* or problems or complications or disturbances or consequences or impairments or dysfunction or function or functioning or functional or abnormalities or dizziness or headache* or dyspnea or fatigue or breath or breathing or lung or respiratory or tachycardia or palpitation* or neuro* or concentration or concentrating or brain fog or cough or ache* or pain* or taste or smell or olfactory or olfaction or gustatory)).mp. and (COVID or COVID-19 or COVID19 or coronavirus* or corona virus* or 2019-nCoV or 19nCoV or 2019nCoV or nCoV or n-CoV or SARS-CoV-2 or SARS-CoV2 or SARSCoV-2 or SARSCoV2 or 2019-novel CoV or Sars-coronavirus2 or novel CoV).tw. 13743

4 ((postcovid or postcorona* or post-COVID or post-COVID-19 or post-COVID19 or post-coronavirus* or post-corona virus*) adj3 (patient or patients or condition* or syndrome or outcomes or symptom* or morbidity or manifestation* or issues or effects or difficulties or challeng* or problems or complications or disturbances or consequences or impairments or dysfunction or function or functioning or functional or abnormalities or dizziness or headache* or dyspnea or fatigue or breath or breathing or lung or respiratory or tachycardia or palpitation* or neuro* or concentration or concentrating or brain fog or cough or ache* or pain* or taste or smell or olfactory or olfaction or gustatory)).mp. 5504

5 (survivor* adj2 (covid or covid-19 or covid19 or coronavirus* or "corona virus*" or 2019-ncov or 19ncov or 2019ncov or ncov or n-cov or sars-cov-2 or sars-cov2 or sarscov-2 or sarscov2 or "2019-novel CoV" or sars-coronavirus2 or "novel CoV")).mp. 1978

6 1 or 2 or 3 or 4 or 5 31109

7 Return to Work/ 4029

8 work capacity/ 0

9 job accommodation/ or work resumption/ 0

10 vocational rehabilitation/ 9881

11 ((work* or job or jobs or employee*) adj3 (modification* or modified or accommodat*)).mp. 5376

12 ("occupational rehab*" or "vocational rehab*" or "workplace intervention*").mp. 4871

13 ((work or worker* or workplace or employ* or occupation or vocational) and (rehab* or telerehab* or mindfulness or "acceptance and commitment therap*" or "relaxation technique*" or "relaxation therap*" or "stop rest pace" or "breathing technique*")).mp. 59451

14 exp long COVID/rh, th [Rehabilitation, Therapy] 48

15 (Return* to work* or "sick* leave*" or "worker* compensation" or WCB or "disability coverage").mp. 34450

16 ((work* or job or jobs or employee* or personnel or professionals) adj3 (perform* or "carry* out" or abilit* or inabilit* or able or return* or reintegrat*)).mp. 79343

17 ((work or worker* or workplace or employ* or job or jobs or personnel or professionals or occupation* or vocational) and ("push-crash cycle" or "boom-bust cycle" or "autonomic dysfunction*" or "post exertional malaise" or PEM or "post exertional symptom exacerbation*" or PESE or "short term disability" or "longterm disability" or "long term disability" or absenteeism or presenteeism)).mp. 13303

18 ((work* or employee* or job* or occupational or vocational) adj3 (performance or limitation* or functioning or efficiency or efficacy or productivity or capacity)).mp. 66632

19 or/7-18 195063

20 6 and 19 790

**Embase <1974 to 2025 January 02> (Ovid interface)**

Date searched: Jan 3, 2025

Results: 1562

<https://login.ezproxy.library.ualberta.ca/login?url=http://ovidsp.ovid.com/ovidweb.cgi?T=JS&NEWS=N&PAGE=main&SHAREDSEARCHID=3R5X921WUe4i7vd2unFMPE12u3yQ1HW1VRxdYbGVF06MJXVtdkPNPCqaj22X3gioY>

1 long COVID/ 9316

2 ((long or (long term not long term care) or long haul* or longterm or longhaul or post acute or postacute or after acute or sequela* or protracted or post-infect* or post-viral or post-discharg* or non-recover* or nonrecover* or PASC or chronic or persist* or linger* or continuing or continual) adj5 (COVID or COVID-19 or COVID19 or coronavirus* or corona virus* or 2019-nCoV or 19nCoV or 2019nCoV or nCoV or n-CoV or SARS-CoV-2 or SARS-CoV2 or SARSCoV-2 or SARSCoV2 or 2019-novel CoV or Sars-coronavirus2 or novel CoV)).mp. 28614

3 ((long or long term or long haul* or longterm or longhaul or ongoing or chronic or lengthy or protracted or persist* or linger* or continuing or continual* or post-acute or postacute or post-viral or post-discharg* or post-infect* or residual) adj2 (outcomes or symptom* or morbidity or manifestation* or issues or effects or difficulties or challeng* or problems or complications or disturbances or consequences or impairments or dysfunction or function or functioning or functional or abnormalities or dizziness or headache* or dyspnea or fatigue or breath or breathing or lung or respiratory or tachycardia or palpitation* or neuro* or concentration or concentrating or brain fog or cough or ache* or pain* or taste or smell or olfactory or olfaction or gustatory)).mp. and (COVID or COVID-19 or COVID19 or coronavirus* or corona virus* or 2019-nCoV or 19nCoV or 2019nCoV or nCoV or n-CoV or SARS-CoV-2 or SARS-CoV2 or SARSCoV-2 or SARSCoV2 or 2019-novel CoV or Sars-coronavirus2 or novel CoV).tw. 27784

4 ((postcovid or postcorona* or post-COVID or post-COVID-19 or post-COVID19 or post-coronavirus* or post-corona virus*) adj3 (patient or patients or condition* or syndrome or outcomes or symptom* or morbidity or manifestation* or issues or effects or difficulties or challeng* or problems or complications or disturbances or consequences or impairments or dysfunction or function or functioning or functional or abnormalities or dizziness or headache* or dyspnea or fatigue or breath or breathing or lung or respiratory or tachycardia or palpitation* or neuro* or concentration or concentrating or brain fog or cough or ache* or pain* or taste or smell or olfactory or olfaction or gustatory)).mp. 7765

5 (survivor* adj2 (covid or covid-19 or covid19 or coronavirus* or "corona virus*" or 2019-ncov or 19ncov or 2019ncov or ncov or n-cov or sars-cov-2 or sars-cov2 or sarscov-2 or sarscov2 or "2019-novel CoV" or sars-coronavirus2 or "novel CoV")).mp. 2665

6 1 or 2 or 3 or 4 or 5 49731

7 Return to Work/ 11050

8 work capacity/ 14400

9 job accommodation/ or work resumption/ 3983

10 vocational rehabilitation/ 9079

11 ((work* or job or jobs or employee*) adj3 (modification* or modified or accommodat*)).mp. 7001

12 ("occupational rehab*" or "vocational rehab*" or "workplace intervention*").mp. 11961

13 ((work or worker* or workplace or employ* or occupation or vocational) and (rehab* or telerehab* or mindfulness or "acceptance and commitment therap*" or "relaxation technique*" or "relaxation therap*" or "stop rest pace" or "breathing technique*")).mp. 76031

14 exp long COVID/rh, th [Rehabilitation, Therapy] 330

15 (Return* to work* or "sick* leave*" or "worker* compensation" or WCB or "disability coverage").mp. 36047

16 ((work* or job or jobs or employee* or personnel or professionals) adj3 (perform* or "carry* out" or abilit* or inabilit* or able or return* or reintegrat*)).mp. 113051

17 ((work or worker* or workplace or employ* or job or jobs or personnel or professionals or occupation* or vocational) and ("push-crash cycle" or "boom-bust cycle" or "autonomic dysfunction*" or "post exertional malaise" or PEM or "post exertional symptom exacerbation*" or PESE or "short term disability" or "longterm disability" or "long term disability" or absenteeism or presenteeism)).mp. 23111

18 ((work* or employee* or job* or occupational or vocational) adj3 (performance or limitation* or functioning or efficiency or efficacy or productivity or capacity)).mp. 96684

19 or/7-18 260184

20 6 and 19 1562

**APA PsycInfo <1806 to December 2024 Week 4>(Ovid interface)**

Date searched: Jan 3, 2025

Results: 120

<https://login.ezproxy.library.ualberta.ca/login?url=http://ovidsp.ovid.com/ovidweb.cgi?T=JS&NEWS=N&PAGE=main&SHAREDSEARCHID=4LFNdsE3eIXqQnjq1OPoQiwJogsaVt8K9jtt7VRCiNclToibZnDYUBWitzzFSTa9d>

1 post-covid-19 conditions/ 372

2 ((long or (long term not long term care) or long haul* or longterm or longhaul or post acute or postacute or after acute or sequela* or protracted or post-infect* or post-viral or post-discharg* or non-recover* or nonrecover* or PASC or chronic or persist* or linger* or continuing or continual) adj5 (COVID or COVID-19 or COVID19 or coronavirus* or corona virus* or 2019-nCoV or 19nCoV or 2019nCoV or nCoV or n-CoV or SARS-CoV-2 or SARS-CoV2 or SARSCoV-2 or SARSCoV2 or 2019-novel CoV or Sars-coronavirus2 or novel CoV)).mp. 1830

3 ((long or long term or long haul* or longterm or longhaul or ongoing or chronic or lengthy or protracted or persist* or linger* or continuing or continual* or post-acute or postacute or post-viral or post-discharg* or post-infect* or residual) adj2 (outcomes or symptom* or morbidity or manifestation* or issues or effects or difficulties or challeng* or problems or complications or disturbances or consequences or impairments or dysfunction or function or functioning or functional or abnormalities or dizziness or headache* or dyspnea or fatigue or breath or breathing or lung or respiratory or tachycardia or palpitation* or neuro* or concentration or concentrating or brain fog or cough or ache* or pain* or taste or smell or olfactory or olfaction or gustatory)).mp. and (COVID or COVID-19 or COVID19 or coronavirus* or corona virus* or 2019-nCoV or 19nCoV or 2019nCoV or nCoV or n-CoV or SARS-CoV-2 or SARS-CoV2 or SARSCoV-2 or SARSCoV2 or 2019-novel CoV or Sars-coronavirus2 or novel CoV).tw. 1637

4 ((postcovid or postcorona* or post-COVID or post-COVID-19 or post-COVID19 or post-coronavirus* or post-corona virus*) adj3 (patient or patients or condition* or syndrome or outcomes or symptom* or morbidity or manifestation* or issues or effects or difficulties or challeng* or problems or complications or disturbances or consequences or impairments or dysfunction or function or functioning or functional or abnormalities or dizziness or headache* or dyspnea or fatigue or breath or breathing or lung or respiratory or tachycardia or palpitation* or neuro* or concentration or concentrating or brain fog or cough or ache* or pain* or taste or smell or olfactory or olfaction or gustatory)).mp. 665

5 (survivor* adj2 (covid or covid-19 or covid19 or coronavirus* or "corona virus*" or 2019-ncov or 19ncov or 2019ncov or ncov or n-cov or sars-cov-2 or sars-cov2 or sarscov-2 or sarscov2 or "2019-novel CoV" or sars-coronavirus2 or "novel CoV")).mp. 291

6 1 or 2 or 3 or 4 or 5 3111

7 reemployment/ 2037

8 job performance/ or employee productivity/ 24908

9 employee absenteeism/ 2516

10 vocational rehabilitation/ 6545

11 ((work* or job or jobs or employee*) adj3 (modification* or modified or accommodat*)).mp. 2271

12 ("occupational rehab*" or "vocational rehab*" or "workplace intervention*").mp. 10323

13 ((work or worker* or workplace or employ* or occupation or vocational) and (rehab* or telerehab* or mindfulness or "acceptance and commitment therap*" or "relaxation technique*" or "relaxation therap*" or "stop rest pace" or "breathing technique*")).mp. 32936

14 (Return* to work* or "sick* leave*" or "worker* compensation" or WCB or "disability coverage").mp. 7717

15 ((work* or job or jobs or employee* or personnel or professionals) adj3 (perform* or "carry* out" or abilit* or inabilit* or able or return* or reintegrat*)).mp. 58273

16 ((work or worker* or workplace or employ* or job or jobs or personnel or professionals or occupation* or vocational) and ("push-crash cycle" or "boom-bust cycle" or "autonomic dysfunction*" or "post exertional malaise" or PEM or "post exertional symptom exacerbation*" or PESE or "short term disability" or "longterm disability" or "long term disability" or absenteeism or presenteeism)).mp. 6123

17 ((work* or employee* or job* or occupational or vocational) adj3 (performance or limitation* or functioning or efficiency or efficacy or productivity or capacity)).mp. 63479

18 ((work* or employee* or job* or occupational or vocational) adj3 (performance or limitation* or functioning or efficiency or efficacy or productivity or capacity)).mp. 63479

19 or/7-18 121500

20 6 and 19 120

**CINAHL Plus with Full Text (EBSCOhost Interface)**

Date searched: Jan 3, 2025

Results: 311

[Search link](https://login.ezproxy.library.ualberta.ca/login?url=https://search.ebscohost.com/login.aspx?direct=true&db=rzh&bquery=((MH+%26quot%3bPost-Acute+COVID-19+Syndrome%26quot%3b)+OR+((long+OR+(long-term+NOT+long-term-care)+OR+long-haul*+OR+longterm+OR+longhaul+OR+post-acute+OR+postacute+OR+after-acute+OR+sequela*+OR+protracted+OR+post-infect*+OR+post-viral+OR+post-discharg*+OR+non-recover*+OR+nonrecover*+OR+PASC+OR+chronic+OR+persist*+OR+linger*+OR+continuing+OR+continual)+N5+(COVID+OR+COVID-19+OR+COVID19+OR+coronavirus*+OR+corona-virus*+OR+2019-nCoV+OR+19nCoV+OR+2019nCoV+OR+nCoV+OR+n-CoV+OR+SARS-CoV-2+OR+SARS-CoV2+OR+SARSCoV-2+OR+SARSCoV2+OR+2019-novel-CoV+OR+Sars-coronavirus2+OR+(novel+AND+CoV)))+OR+(((long+OR+long-term+OR+long-haul*+OR+longterm+OR+longhaul+OR+ongoing+OR+chronic+OR+lengthy+OR+protracted+OR+persist*+OR+linger*+OR+continuing+OR+continual*+OR+post-acute+OR+postacute+OR+post-viral+OR+post-discharg*+OR+post-infect*+OR+residual)+N2+(outcomes+OR+symptom*+OR+morbidity+OR+manifestation*+OR+issues+OR+effects+OR+difficulties+OR+challeng*+OR+problems+OR+complications+OR+disturbances+OR+consequences+OR+impairments+OR+dysfunction+OR+function+OR+functioning+OR+functional+OR+abnormalities+OR+dizziness+OR+headache*+OR+dyspnea+OR+fatigue+OR+breath+OR+breathing+OR+lung+OR+respiratory+OR+tachycardia+OR+palpitation*+OR+neuro*+OR+concentration+OR+concentrating+OR+brain-fog+OR+cough+OR+ache*+OR+pain*+OR+taste+OR+smell+OR+olfactory+OR+olfaction+OR+gustatory))+AND+(COVID+OR+COVID-19+OR+COVID19+OR+coronavirus*+OR+corona-virus*+OR+2019-nCoV+OR+19nCoV+OR+2019nCoV+OR+nCoV+OR+n-CoV+OR+SARS-CoV-2+OR+SARS-CoV2+OR+SARSCoV-2+OR+SARSCoV2+OR+2019-novel-CoV+OR+Sars-coronavirus2+OR+(novel+AND+CoV)))+OR+((postcovid+OR+postcorona*+OR+post-COVID+OR+post-COVID-19+OR+post-COVID19+OR+post-coronavirus*+OR+post-corona-virus*+OR+post-acute-covid*)+N3+(patient+OR+patients+OR+condition*+OR+syndrome+OR+outcomes+OR+symptom*+OR+morbidity+OR+manifestation*+OR+issues+OR+effects+OR+difficulties+OR+challeng*+OR+problems+OR+complications+OR+disturbances+OR+consequences+OR+impairments+OR+dysfunction+OR+function+OR+functioning+OR+functional+OR+abnormalities+OR+dizziness+OR+headache*+OR+dyspnea+OR+fatigue+OR+breath+OR+breathing+OR+lung+OR+respiratory+OR+tachycardia+OR+palpitation*+OR+neuro*+OR+concentration+OR+concentrating+OR+brain-fog+OR+cough+OR+ache*+OR+pain*+OR+taste+OR+smell+OR+olfactory+OR+olfaction+OR+gustatory))+OR+(survivor*+N2+(covid+OR+covid-19+OR+covid19+OR+coronavirus*+OR+%26quot%3bcorona+virus*%26quot%3b+OR+2019-ncov+OR+19ncov+OR+2019ncov+OR+ncov+OR+n-cov+OR+sars-cov-2+OR+sars-cov2+OR+sarscov-2+OR+sarscov2+OR+%26quot%3b2019-novel+CoV%26quot%3b+OR+sars-coronavirus2+OR+%26quot%3bnovel+CoV%26quot%3b)))+AND+(((MH+%26quot%3bJob+Accommodation%26quot%3b)+OR+(MH+%26quot%3bJob+Re-Entry%26quot%3b)+OR+(MH+%26quot%3bJob+Performance%26quot%3b))+OR+(((work*+OR+job+OR+jobs+OR+employee*)+N3+(modification*+OR+modified+OR+accommodat*))+OR+%26quot%3boccupational+rehab*%26quot%3b+OR+%26quot%3bvocational+rehab*%26quot%3b+OR+%26quot%3bworkplace+intervention*%26quot%3b+OR+%26quot%3bReturn*+to+work*%26quot%3b+OR+%26quot%3bsick*+leave*%26quot%3b+OR+%26quot%3bworker*+compensation%26quot%3b+OR+WCB+OR+%26quot%3bdisability+coverage%26quot%3b)+OR+(((work+OR+worker*+OR+workplace+OR+employ*+OR+occupation+OR+vocational)+AND+(rehab*+OR+telerehab*+OR+mindfulness+OR+%26quot%3bacceptance+and+commitment+therap*%26quot%3b+OR+%26quot%3brelaxation+technique*%26quot%3b+OR+%26quot%3brelaxation+therap*%26quot%3b+OR+%26quot%3bstop+rest+pace%26quot%3b+OR+%26quot%3bbreathing+technique*%26quot%3b)))+OR+(((work*+OR+job+OR+jobs+OR+employee*+OR+personnel+OR+professionals)+N3+(perform*+OR+%26quot%3bcarry*+out%26quot%3b+OR+abilit*+OR+inabilit*+OR+able+OR+return*+OR+reintegrat*)))+OR+(((work+OR+worker*+OR+workplace+OR+employ*+OR+job+OR+jobs+OR+personnel+OR+professionals+OR+occupation*+OR+vocational)+AND+(%26quot%3bpush-crash+cycle%26quot%3b+OR+%26quot%3bboom-bust+cycle%26quot%3b+OR+%26quot%3bautonomic+dysfunction*%26quot%3b+OR+%26quot%3bpost+exertional+malaise%26quot%3b+OR+PEM+OR+%26quot%3bpost+exertional+symptom+exacerbation*%26quot%3b+OR+PESE+OR+%26quot%3bshort+term+disability%26quot%3b+OR+%26quot%3blongterm+disability%26quot%3b+OR+%26quot%3blong+term+disability%26quot%3b+OR+absenteeism+OR+presenteeism)))+OR+(((work*+OR+employee*+OR+job*+OR+occupational+OR+vocational)+N3+(performance+OR+limitation*+OR+functioning+OR+efficiency+OR+efficacy+OR+productivity+OR+capacity))))&type=1&searchMode=And&site=ehost-live&scope=site)

S1: (MH "Post-Acute COVID-19 Syndrome") OR ( ((long or (long-term not long-term-care) or long-haul* or longterm or longhaul or post-acute or postacute or after-acute or sequela* or protracted or post-infect* or post-viral or post-discharg* or non-recover* or nonrecover* or PASC or chronic or persist* or linger* or continuing or continual) N5 (COVID or COVID-19 or COVID19 or coronavirus* or corona-virus* or 2019-nCoV or 19nCoV or 2019nCoV or nCoV or n-CoV or SARS-CoV-2 or SARS-CoV2 or SARSCoV-2 or SARSCoV2 or 2019-novel-CoV or Sars-coronavirus2 or novel CoV)) ) OR ( ((long or long-term or long-haul* or longterm or longhaul or ongoing or chronic or lengthy or protracted or persist* or linger* or continuing or continual* or post-acute or postacute or post-viral or post-discharg* or post-infect* or residual) N2 (outcomes or symptom* or morbidity or manifestation* or issues or effects or difficulties or challeng* or problems or complications or disturbances or consequences or impairments or dysfunction or function or functioning or functional or abnormalities or dizziness or headache* or dyspnea or fatigue or breath or breathing or lung or respiratory or tachycardia or palpitation* or neuro* or concentration or concentrating or brain-fog or cough or ache* or pain* or taste or smell or olfactory or olfaction or gustatory)) AND (COVID or COVID-19 or COVID19 or coronavirus* or corona-virus* or 2019-nCoV or 19nCoV or 2019nCoV or nCoV or n-CoV or SARS-CoV-2 or SARS-CoV2 or SARSCoV-2 or SARSCoV2 or 2019-novel-CoV or Sars-coronavirus2 or novel CoV) ) OR ((postcovid or postcorona* or post-COVID or post-COVID-19 or post-COVID19 or post-coronavirus* or post-corona-virus* or post-acute-covid*) N3 (patient or patients or condition* or syndrome or outcomes or symptom* or morbidity or manifestation* or issues or effects or difficulties or challeng* or problems or complications or disturbances or consequences or impairments or dysfunction or function or functioning or functional or abnormalities or dizziness or headache* or dyspnea or fatigue or breath or breathing or lung or respiratory or tachycardia or palpitation* or neuro* or concentration or concentrating or brain-fog or cough or ache* or pain* or taste or smell or olfactory or olfaction or gustatory)) OR ( survivor* N2 ( covid OR covid-19 OR covid19 OR coronavirus* OR "corona virus*" OR 2019-ncov OR 19ncov OR 2019ncov OR ncov OR n-cov OR sars-cov-2 OR sars-cov2 OR sarscov-2 OR sarscov2 OR "2019-novel CoV" OR sars-coronavirus2 OR "novel CoV" ) )

S2: (MH "Job Accommodation") or (MH "Job Re-Entry") OR (MH "Job Performance")

S3: ((work* or job or jobs or employee*) N3 (modification* or modified or accommodat*)) ) OR "occupational rehab*" or "vocational rehab*" or "workplace intervention*" or "Return* to work*" or "sick* leave*" or "worker* compensation" or WCB or "disability coverage"

S4 ((work or worker* or workplace or employ* or occupation or vocational) and (rehab* or telerehab* or mindfulness or "acceptance and commitment therap*" or "relaxation technique*" or "relaxation therap*" or "stop rest pace" or "breathing technique*"))

S5 ((work* or job or jobs or employee* or personnel or professionals) N3 (perform* or "carry* out" or abilit* or inabilit* or able or return* or reintegrat*))

S6 ((work or worker* or workplace or employ* or job or jobs or personnel or professionals or occupation* or vocational) and ("push-crash cycle" or "boom-bust cycle" or "autonomic dysfunction*" or "post exertional malaise" or PEM or "post exertional symptom exacerbation*" or PESE or "short term disability" or "longterm disability" or "long term disability" or absenteeism or presenteeism))

S7 ((work* or employee* or job* or occupational or vocational) N3 (performance or limitation* or functioning or efficiency or efficacy or productivity or capacity))

S8 S1 AND (S2 OR S3 OR S4 OR S5 OR S6 OR S7)

**Cochrane Library** - Trials database only (Wiley Interface)

Date searched: Jan 3, 2025

Results: ( Trials =161 )

#1 [mh ^"Post-Acute COVID-19 Syndrome"]

#2 ((long or long-term or long-haul or long-hauler or longterm or longhaul or post-acute or postacute or after-acute or sequela* or protracted or post-infection or post-viral or post-discharge or non-recovered or nonrecover* or PASC or chronic or persist* or linger* or continuing or continual) NEAR/5 (COVID or COVID-19 or COVID19 or coronavirus* or corona-virus or "2019-nCoV" or "19nCoV" or "2019nCoV" or nCoV or n-CoV or SARS-CoV-2 or SARS-CoV2 or SARSCoV-2 or SARSCoV2 or "2019-novel-CoV" or Sars-coronavirus2 or novel-CoV)):ti,ab,kw

#3 ((long or long-term or long-haul or long-hauler or longterm or longhaul or ongoing or chronic or lengthy or protracted or persist* or linger* or continuing or continual* or post-acute or postacute or post-viral or post-discharge or post-infection or residual) NEAR/2 (outcomes or symptom* or morbidity or manifestation* or issues or effects or difficulties or challeng* or problems or complications or disturbances or consequences or impairments or dysfunction or function or functioning or functional or abnormalities or dizziness or headache* or dyspnea or fatigue or breath or breathing or lung or respiratory or tachycardia or palpitation* or neuro* or concentration or concentrating or brain-fog or cough or ache* or pain* or taste or smell or olfactory or olfaction or gustatory)):ti,ab,kw and (COVID or COVID-19 or COVID19 or coronavirus* or corona-virus or "2019-nCoV" or "19nCoV" or "2019nCoV" or nCoV or n-CoV or SARS-CoV-2 or SARS-CoV2 or SARSCoV-2 or SARSCoV2 or "2019-novel-CoV" or Sars-coronavirus2 or novel-CoV):ti,ab,kw

#4 ((postcovid or postcorona* or post-COVID or post-COVID-19 or post-COVID19 or post-coronavirus or post-corona-virus or post-acute-covid) NEAR/3 (patient or patients or condition* or syndrome or outcomes or symptom* or morbidity or manifestation* or issues or effects or difficulties or challeng* or problems or complications or disturbances or consequences or impairments or dysfunction or function or functioning or functional or abnormalities or dizziness or headache* or dyspnea or fatigue or breath or breathing or lung or respiratory or tachycardia or palpitation* or neuro* or concentration or concentrating or brain-fog or cough or ache* or pain* or taste or smell or olfactory or olfaction or gustatory)):ti,ab,kw

#5 ( survivor* NEAR/2 (COVID or COVID-19 or COVID19 or coronavirus* or corona-virus or "2019-nCoV" or "19nCoV" or "2019nCoV" or nCoV or n-CoV or SARS-CoV-2 or SARS-CoV2 or SARSCoV-2 or SARSCoV2 or "2019-novel-CoV" or Sars-coronavirus2 or novel-CoV)):ti,ab,kw

#6 (#1 OR #2 OR #3 OR #4 OR #5)

#7 ((work* or job or jobs or employee*) NEAR/3 (modification* or modified or accommodat*)):ti,ab,kw

#8 ("occupational rehabilitation" or "vocational rehabilitation" or "workplace intervention"):ti,ab,kw

#9 ((work or worker* or workplace or employ* or occupation or vocational) and (rehab* or telerehab* or mindfulness or "acceptance and commitment therapy" or "relaxation technique" or "relaxation therapy" or "stop rest pace" or "breathing technique")):ti,ab,kw

#10 [mh ^"Post-Acute COVID-19 Syndrome"/rh,th]

#11 (Return-to-work or "sick leave" or (worker* NEXT compensation) or WCB or "disability coverage"):ti,ab,kw

#12 ((work* or job or jobs or employee* or personnel or professionals) NEAR/3 (perform* or "carry out" or "carrying out" or abilit* or inabilit* or able or return* or reintegrat*)):ti,ab,kw

#13 ((work or worker* or workplace or employ* or job or jobs or personnel or professionals or occupation* or vocational) and ("push-crash cycle" or "boom-bust cycle" or "autonomic dysfunction" or "post exertional malaise" or PEM or "post exertional symptom exacerbation" or PESE or "short term disability" or "longterm disability" or "long term disability" or absenteeism or presenteeism)):ti,ab,kw

#14 ((work* or employee* or job* or occupational or vocational) NEAR/3 (performance or limitation* or functioning or efficiency or efficacy or productivity or capacity)):ti,ab,kw

#15 #7 OR #8 OR #9 OR #10 OR #11 OR #12 OR #13 OR #14

#16 #6 AND #15

**Scopus (Advanced Search)**

Date searched: Jan 3, 2025

Results: 1370

TITLE-ABS-KEY ( ((long or (long-term AND NOT long-term-care) or long-haul* or longterm or longhaul or post-acute or postacute or after-acute or sequela* or protracted or post-infect* or post-viral or post-discharg* or non-recover* or nonrecover* or PASC or chronic or persist* or linger* or continuing or continual) W/5 (COVID or COVID-19 or COVID19 or coronavirus* or corona-virus* or 2019-nCoV or 19nCoV or 2019nCoV or nCoV or n-CoV or SARS-CoV-2 or SARS-CoV2 or SARSCoV-2 or SARSCoV2 or 2019-novel-CoV or Sars-coronavirus2 or "novel CoV")) OR ( ((long or long-term or long-haul* or longterm or longhaul or ongoing or chronic or lengthy or protracted or persist* or linger* or continuing or continual* or post-acute or postacute or post-viral or post-discharg* or post-infect* or residual) W/2 (outcomes or symptom* or morbidity or manifestation* or issues or effects or difficulties or challeng* or problems or complications or disturbances or consequences or impairments or dysfunction or function or functioning or functional or abnormalities or dizziness or headache* or dyspnea or fatigue or breath or breathing or lung or respiratory or tachycardia or palpitation* or neuro* or concentration or concentrating or brain-fog or cough or ache* or pain* or taste or smell or olfactory or olfaction or gustatory)) AND (COVID or COVID-19 or COVID19 or coronavirus* or corona-virus* or 2019-nCoV or 19nCoV or 2019nCoV or nCoV or n-CoV or SARS-CoV-2 or SARS-CoV2 or SARSCoV-2 or SARSCoV2 or 2019-novel-CoV or Sars-coronavirus2 or "novel CoV") ) OR ((postcovid or postcorona* or post-COVID or post-COVID-19 or post-COVID19 or post-coronavirus* or post-corona-virus* or post-acute-covid*) W/3 (patient or patients or condition* or syndrome or outcomes or symptom* or morbidity or manifestation* or issues or effects or difficulties or challeng* or problems or complications or disturbances or consequences or impairments or dysfunction or function or functioning or functional or abnormalities or dizziness or headache* or dyspnea or fatigue or breath or breathing or lung or respiratory or tachycardia or palpitation* or neuro* or concentration or concentrating or brain-fog or cough or ache* or pain* or taste or smell or olfactory or olfaction or gustatory)) OR ( survivor* W/2 ( covid OR covid-19 OR covid19 OR coronavirus* OR "corona virus*" OR 2019-ncov OR 19ncov OR 2019ncov OR ncov OR n-cov OR sars-cov-2 OR sars-cov2 OR sarscov-2 OR sarscov2 OR "2019-novel CoV" OR sars-coronavirus2 OR "novel CoV" ) ))

AND

TITLE-ABS-KEY(((work* or job or jobs or employee*) W/3 (modification* or modified or accommodat*)) OR "occupational rehab*" or "vocational rehab*" or "workplace intervention*" or "Return* to work*" or "sick* leave*" or "worker* compensation" or WCB or "disability coverage" OR ((work or worker* or workplace or employ* or occupation or vocational) and (rehab* or telerehab* or mindfulness or "acceptance and commitment therap*" or "relaxation technique*" or "relaxation therap*" or "stop rest pace" or "breathing technique*")) OR ((work* or job or jobs or employee* or personnel or professionals) W/3 (perform* or "carry* out" or abilit* or inabilit* or able or return* or reintegrat*)) OR ((work or worker* or workplace or employ* or job or jobs or personnel or professionals or occupation* or vocational) and ("push-crash cycle" or "boom-bust cycle" or "autonomic dysfunction*" or "post exertional malaise" or PEM or "post exertional symptom exacerbation*" or PESE or "short term disability" or "longterm disability" or "long term disability" or absenteeism or presenteeism)) OR ((work* or employee* or job* or occupational or vocational) W/3 (performance or limitation* or functioning or efficiency or efficacy or productivity or capacity)))

Grey literature search

**Government of Canada Publications** (<https://www.publications.gc.ca/site/eng/home.html>) - Searched Jan 6, 2025 (post-covid; long-covid) (3 potentially useful results, 0 initial results)

**Custom Search Engine for Canadian Public Health Information** (<https://www.ophla.ca/p/customsearchcanada.html>)

Searched Jan 6, 2025 and November 7, 2023

"return to work" long-covid OR post-covid-syndrome OR post-covid-condition OR post-covid-19-condition OR post-covid-19-syndrome (retrieved 24 results in Nov 2023, 10 updated on Jan 6 2025)

**Custom Search Engine for US State Government Information (**[**https://www.ophla.ca/p/customsearchusstates.html**](https://www.ophla.ca/p/customsearchusstates.html)**)**

Searched Jan 6, 2025 and November 7, 2023

"return to work" long-covid OR post-covid-syndrome OR post-covid-condition OR post-covid-19-condition OR post-covid-19-syndrome (retrieved 11 results in Nov 2023, 4 updated on Jan 6 2025)

**MedRxiv (**[**https://www.medrxiv.org/**](https://www.medrxiv.org/)**) (USE ADVANCED SEARCH,** Searched in title/abstract**)**

Searched November 7, 2023 and Jan 6, 2025

(total 7 results in Nov 2023 and total of 10 in Jan 2025)

Long-covid return-to-work (all terms) - 5 results in Nov 2023, 10 additional results in Jan 2025

post-covid return-to-work (all terms) - 3 results in Nov 2023, 8 additional results in Jan 2025

**Bielefeld Academic Search Engine (BASE) (https://www.base-search.net/)**

Searched November 7, 2023 and Jan 6, 2025

Searched "all fields" and "verbatim search"

"Return to work" "long covid" - 75 results in Nov 2023, 51 additional hits in Jan 2025

"Return to work" "post covid" 110 results in Nov 2023, 46 additional hits in Jan 2025

**OAIster (oaister.on.worldcat.org) - Use Advanced search**

Searched November 7, 2023 (Total 12 items downloaded in Nov 2023- didn't download duplicates) and Jan 6 2025 (Total of 6 new items downloaded in Jan 2025 - didn't download duplicates)

"Return to work" long-covid

"Return to work" post-covid

**Google (Advanced search) (searched with an incognito browser):**

Searched Nov 7, 2023 and Jan 6, 2025

"return to work" long-covid OR post-covid-syndrome OR post-covid-condition OR post-covid-19-condition OR post-covid-19-syndrome filetype:pdf (review 168 results in Nov 2023; review 50 updated results in Jan 2025)

"return to work" long-covid OR post-covid-syndrome OR post-covid-condition OR post-covid-19-condition OR post-covid-19-syndrome -filetype:pdf -site:pubmed.ncbi.nlm.nih.gov -Oxford -elsevier -science-direct -sage -wiley (review first 100 results in Nov 2023; review 50 updated results in Jan 2025)

"Workers Compensation" long-covid OR post-covid-syndrome OR post-covid-condition OR post-covid-19-condition OR post-covid-19-syndrome (review first 50 results in Nov 2023; review 50 updated results in Jan 2025)

**These websites were scanned for any relevant documents:**

**Alberta Health Services "Recovery & Rehabilitation After COVID-19: Resources for Health Professionals**

[**https://www.albertahealthservices.ca/topics/Page17540.aspx**](https://www.albertahealthservices.ca/topics/Page17540.aspx)

**WHO websites relevant to long covid**

Rehabilitation and Covid-19 <https://www.who.int/teams/noncommunicable-diseases/covid-19/rehabilitation>

Post covid condition

<https://www.who.int/teams/health-care-readiness/post-covid-19-condition>

High-level meeting on post-COVID conditions (‎long COVID)‎: a virtual meeting hosted by the WHO Regional Office for Europe, 19 March 2021

<https://www.who.int/europe/publications/i/item/WHO-EURO-2021-2410-42165-58100>
