# Supplementary material for: Return-to-work for people living with long COVID: A scoping review of interventions and recommendations
Source: PLoS One. 2025 Oct 15;20(10):e0321891. doi: 10.1371/journal.pone.0321891 (PMC12527184; doi:10.1371/journal.pone.0321891)
Supplement: S1 Table — (DOCX) [file pone.0321891.s002.docx]

**Table S1. Descriptive Characteristics of Intervention Studies**

| **Author** | **Year published** | **Country** | **Study objectives** | **Design** | **Sample size, n** | **Female, n** | **Age, years** | **Time since Covid-19**  **(months)** | **Study duration** | **Inclusion criteria** |
| --- | --- | --- | --- | --- | --- | --- | --- | --- | --- | --- |
| Frisk et al. 2023: A safe and effective micro-choice-based rehabilitation for patients with Long COVID:  Results from a quasi-experimental study  Altmann et al 2023. Pulmonary recovery directly after COVID-19 and in Long COVID  Brehon et al. 2022 Return-to-Work Following Occupational Rehabilitation for Long COVID: Descriptive Cohort Study  Sathyamoorthy et al. 2022 Enhanced external counterpulsation for management of symptoms associated with Long COVID  García-Molina, A. et al., (2022). Neuropsychological rehabilitation for Post-COVID-19 Syndrome: Results of a clinical program and six-month follow up.  Ghali, A., et al. The relevance of pacing strategies in managing symptoms of Post-COVID-19 Syndrome.    Oka T. (2023). A patient who recovered from Post-COVID Myalgic Encephalomyelitis/Chronic Fatigue Syndrome: a case report.    Tanguay, P., et al., (2023). Post-Exertional Malaise may persist in Long COVID despite learning STOP-REST-PACE.  Derksen, C., et al. (2023). Longitudinal Evaluation of an Integrated Post-COVID-19/Long COVID Management Program Consisting of Digital Interventions and Personal Support: Randomized Controlled Trial.  Hasenoehrl, T., et al., (2023). Post-COVID: effects of physical exercise on functional status and work ability in health care personnel.  Müller, K., et al., (2023). Impact of Rehabilitation on Physical and Neuropsychological Health of Patients Who Acquired COVID-19 in the Workplace.  Garbsch, R. et al. (2024). Sex-specific differences of cardiopulmonary fitness and pulmonary function in exercise-based rehabilitation of patients with long-term post-COVID-19 syndrome.    Frisk, B., et al. (2025). Sustained improvements in sick leave, fatigue and functional status following a concentrated micro-choice-based treatment for patients with Long COVID: A 1 year prospective uncontrolled study.  Müller, K., et al. (2024). Long-term course and factors influencing work ability and return to work in post-COVID patients 12 months after inpatient rehabilitation.  Nerli, T. F., et al. (2024). Brief Outpatient Rehabilitation Program for Post-COVID-19 Condition: A Randomized Clinical Trial.  Schmid, S., et al. (2024). Effects of an integrative multimodal inpatient program on fatigue and work ability in patients with Post-COVID Syndrome-a prospective observational study.  Uswatte, G., et al. (2024). Long COVID Brain Fog Treatment: Findings from a Pilot Randomized Controlled Trial of Constraint-Induced Cognitive Therapy.  Wagner, B., Steiner, M., Markovic, L., & Crevenna, R. (2022). Successful application of pulsed electromagnetic fields in a patient with Post-COVID-19 fatigue: a case report.  Kupferschmitt, A., et al., (2023). First results from post-COVID inpatient rehabilitation. | 2023  2023  2022  2022  2022  2023  2023  2023  2023  2023  2023  2024  2025  2024  2024  2024  2024  2022  2023 | Norway  Germany  Canada  Texas, USA  Barcelona, Spain  France  Japan  Canada  Germany  Vienna, Austria  Germany  Germany  Norway  Germany  Norway  Germany  United States of America  Germany  Germany | To assess safety, acceptability, potential changes in fatigue, sick leave, functional level, dyspnea, and exercise capacity from pre-treatment to 3 months follow-up and to explore predictors for change in fatigue    To present data on pulmonary impairment, pulmonary recovery and outcome comparing patients admitted to a specific COVID-19 rehabilitation program directly after COVID-19 infection with patients long after COVID-19 infection.  This study describes the characteristics and outcomes of workers who participated in a Long COVID occupational rehabilitation program.  Enhanced external counterpulsation (EECP) as a possible therapy for Long COVID.  The objective of this study is to analyse the results of a neuropsychological rehabilitation programme aimed at patients with Post-COVID-19 Syndrome. The effects of treatment were evaluated by applying an evaluation protocol before and after the intervention, and at 6 months after treatment.  To assess the effectiveness of pacing strategies in managing the symptoms of patients with Post-COVID-19 Syndrome, especially in terms of reducing fatigue levels and preventing Post-Exertional Malaise occurrence.  To provide a report on a woman with Long COVID who was infected with SARS-CoV-2 and developed severe fatigue and other debilitating problems that persisted for more than 6 months, thus satisfying the diagnostic criteria for ME/CFS. She recovered after treatment for her ME/CFS that was based on multiple perspectives.    1) To observe Post-Exertional Malaise over 12 weeks of telerehabilitation based on the STOP-REST-PACE approach. (2) To describe the changes in health-related quality of life, respiratory symptoms, fatigue and return to work.    This research aimed to test whether patients who received a health care facilitation program including medical internet support from human personal pilots and digital interventions (intervention group [IG] and active control group [ACG]) would experience fewer symptoms and have higher work ability and social participation than an untreated comparison group. The second objective was to compare the impact of a diagnostic assessment, and digital interventions tailored to patients’ personal capacity (IG) with that of only personal support and digital interventions targeting the main symptoms (ACG).    To assess the effects of physical exercise on post-COVID-19 fatigue and other associated symptoms, by conducting an exercise intervention trial with COVID-19 surviving Health Care Workers working at a large COVID-19 hospital in Vienna.    To examine the changes in physical and neuropsychological health and work ability after inpatient rehabilitation of 127 patients who acquired COVID-19 in the workplace.  To compare the effects of exercise-based rehabilitation on cardiopulmonary exercise capacity, pulmonary function, and (health-related) outcomes including fatigue  This study presents 12-month follow up data from the same cohort previously reported in the 3-month results. The aims were to evaluate patients’ satisfaction with the treatment, illness perception and patient activation as well as potential changes in sick leave, functional levels, fatigue, dyspnea, and exercise capacity from baseline to the 12- month follow-up after a concentrated micro-choice-based intervention in patients with Long COVID.  To address the impact of rehabilitation on work ability and return to work in Post-COVID patients, evaluating work ability and return to work before rehabilitation and 12 months after rehabilitation. Additionally, the study seeks to analyse group differences in work ability and return to work regarding the participation in aftercare interventions until 12 months after rehabilitation to identify potential associations. Also aimed to identify physical and neuropsychological health factors that influence work ability and return to work 12 months post-rehabilitation.  To assess the effectiveness of a brief outpatient rehabilitation program based on a cognitive and behavioral approach for patients with Post COVID-19 condition  To evaluate the effects of an integrative multimodal inpatient program on fatigue and work ability in patients with Post‐COVID Syndrome    This study’s objectives were to evaluate feasibility and efficacy, provisionally, of a new rehabilitation approach, Constraint-Induced Cognitive Therapy (CICT), for post-COVID-19 cognitive sequelae.  The objective of the case report was to evaluate the use of pulsed electromagnetic fields as a treatment for fatigue in a 55-year-old female experiencing persistent weakness, exhaustion, cognitive difficulties, and exertional dyspnea 6.5 months after a SARS-CoV-2 infection.  This paper compares the psychological stress of post-COVID patients and their course in rehabilitation to psychosomatic and psychocardiological patients. | Quasi- experimental longitudinal study  Quasi-experimental  Descriptive Cohort Study  Retrospective Cohort Study  Quasi-experimental  Retrospective cohort study  Case report  Observational prospective cohort study  Partially Randomized Control Trial (RCT)  Exercise Intervention trial  Longitudinal observational study  Prospective observational cohort study  Prospective Interventional Study  Prospective longitudinal observational study    Pragmatic randomized clinical trial  Prospective observational study  Pilot Randomized Control Trial with unblinded, open-label, parallel-arm, partial-crossover design  Case Report  Observational study | 78  21 (21 directly after infection and 21 Long COVID)  81  16  123 selected and 91 underwent intervention  86  1  34 included and 30 completed rehabilitation  1020  28  127  145  78  114  314  64  14  7 in CICT  7 in usual treatment  1  Psychosomatic subsample = 49  Post COVID subsample = 51  Psychocardiological subsample = 50 | 64 (82%)  40% of Acute COVID-19 and 60% of Long COVID patients  52 (64%)  12 (75%)  72 (58.5%) overall and 55 (60.4%) underwent intervention    70 (81.4%)  1  27 (79%)  763 (74.8%)  22 (79%)  97 (76.4%)  52 (35.9%)  64 (82%)  86 (75%)  225 (72%)  70.31%  10 (71.43%)  1  Psychosomatic = 77.6%  Post COVID = 76.5%  Psychocardiological = 76.0% | 40.3 +/- 12.0  Long  COVID patient mean 3 years younger than acute COVID-19 patients  Mean age 48.9 years  Mean age 53.8 +/- 15.3 years  51.02 +/- 12.4 years  41 (ranging from 31-50)  55  47.0 +/- 8.8  45.3 +/- 13.23  45.8  +/-  11.0  50.62  47.1 +/-  12.7 (female)  52.0 +/-  9.1 (male)  40.3 +/- 12.0  50.54 +/-10.85  43 +/- 12  44.1 +/- 11.44 years  50.7 +/- 12.8  55 years old  Psychosomatic = 50.82 +/- 9.37  Post COVID = 51.29 +/- 9.89  Psychocardiological = 54.84 +/-7.70 | 10.2 +/- 4.8  Range from 4-20 months  Mean = 10.1 months  165.2 days  8.3 +/- 3.6 months  7.7 +/- 3.45 months  12 months (7-17 range)  8 months  146.0 +/- 96.0 days  N/A  6.1 +/- 3.1 months  412.90 +/- 143.61 days  285.5 +/-  140.6 days (female)  248.8 +/- 112.0 days  10.2 +/- 4.8  412.90 +/- 143.61 days  Usual care group = 266 +/- 185 days  Intervention group = 244 +/-154 days  ~22.84 weeks +/-23.71  10.1 +/- 6.5 months  6.5 months  At least 3 months | 3-day concentrated rehabilitation with 7-day and 3-month follow-up  4-5 weeks  49.9 days  Modified 15 treatment sessions or full 35 sessions    8-week and 6-month follow-up  Median 10 months range 6-13  21 weeks  12 weeks  10-12 weeks  8 weeks  Mean 28.77 days (9-42 days)  ~4 weeks (28.8 +/- 6.1 days) inpatient rehabilitation  3-day concentrated rehabilitation with 7-day  3- and 12-month follow-up  Mean 28.77 days (9-42 days)  With 6 month and 12 month follow up  2 to 8 sessions over 2 to 6 weeks  12.63 +/- 1.41 days  36 hours of training over 2 to 7 weeks dependent on participant needs  10 sessions over 5 weeks  Not reported | - Adults 18-67 years of age  - with Long COVID with confirmed infection and persisting symptoms for at least 3 months  - diseases where physical activity is not contraindicated  - had to be fluent in oral and written Norwegian  - sufficient digital competence for online questionnaires  - most individuals not hospitalized in acute phase  Patients undergone Cardiopulmonary exercise testing  The multidisciplinary program includes occupational, physical, and exercise therapy, along with psychology, nursing, and medical interventions as needed. It offers psychoeducation for managing Long COVID symptoms, pacing, energy conservation, and breathing strategies. Exercise is prescribed as tolerated to avoid post-exertional malaise. Programs are delivered in person, via telerehabilitation, or both, based on individual needs. The primary goal is return to work, providing advice on work activities, exploring modified duties, and negotiating with employers.    All patients needed to have a lab documented positive COVID-19 diagnosis prior to March 1, 2021, and referred for the management of Long COVID- related symptoms.  1) age of 18 years or older at the time of the infection, 2) PCR-confirmed SARS-CoV-2 infection, and  3) meeting criteria for post—COVID-19 syndrome.  ***Excluded:***  1) neurological and/or psychiatric dis- ease prior to SARS-CoV-2 infection,  2) cognitive impairment prior to COVID-19, and  3) severe neurological disease secondary to the virus (e.g., encephalopathy, stroke, myelopathy, or polyradiculoneuropathies).  ***Included***: those who met the World Health Organization Post COVID Syndrome definition  ***Excluded:*** patients who had medical records with missing or incomplete data, especially about pacing adherence and patients who were lost to follow-up.  ME/CFS after SARs-CoV-2 infection  ***Inclusion:*** (1) adults (>18 years old) with persistent symptoms for longer than four weeks after confirmed or suspected COVID-19 (prior to WHO definition); (2) scoring below 70/100 points on EuroQol Visual Analogue Scale (EQ-VAS) at entry into study; (3) having access to a computer or tablet to run the telerehabilitation platform and (4) having at least three out of 11 pre-identified impairments (ICF) and symptoms: dyspnea at rest, dyspnea on exertion, pain, presence of fatigue, decreased strength in upper body, decreased strength in lower body, limitations at work, limitations at usual activities, limitations to interact with others, phlegm and cough  ***Exclusion:*** participants already receiving rehabilitation services at the time of recruitment.    The participants had ≥3 (of 14) Post-Acute COVID-19 Syndrome symptoms with severity ≥2 on a scale from 0 (no problem) to 3 (extreme problem).  - Symptoms could not be attributed to other causes by the participants.  - Symptoms were new or exacerbated after a SARS-CoV-2 infection.  - The SARS-CoV-2 infection had been diagnosed more than 4 weeks ago.  - The participants had not fully or substantially recovered from their Post-Acute COVID-19 Syndrome symptoms.  - The participants suffered from impairments in their daily life. - between 18 and 60 years of age - no previous treatment  - did not work in health care  - had low or no care of degree according to Germany standards  ***Inclusion criteria*** were employment at the General Hospital of Vienna, Austria, regardless of position and status Post COVID-19 infection. ***Exclusion criteria*** were preexisting contraindications to aerobic exercise and resistance training as well as insufficient language skills.    Post- Acute phase (>3 months since infection) then screening for eligibility done by nurse with no specifics provided in study    History of at least one COVID-19 infection (positive PCR test at the time of infection), and ongoing or newly expressed performance deficits lasting for at least 3 months prior to recruitment    ***Inclusion criteria:*** Patients were referred to the Department of Thoracic Medicine, by their general practitioner, or other physicians. Patients aged 18 to 67 years, presenting with Long COVID, defined as confirmed SARS-CoV-2 infection with persistent symptoms lasting at least 3 months from the onset of the initial infection and persisting for a minimum of 2 months, were eligible for inclusion. To get access to the specialist healthcare, these symptoms had to have resulted in impaired daily functioning and could not be explained by alternative diagnosis. Participants needed to be fluent in oral and written Norwegian and possess adequate digital skills to complete online questionnaires. ***Exclusion criteria:*** patients who had improvements in symptoms while awaiting treatment were excluded. individuals with other medical conditions that contraindicated physical activity were not offered participation.  Post- Acute phase (>3 months since infection) then screening for eligibility done by nurse with no specifics provided in study  Self-referral or physician referral. ***Inclusion criteria*** (1) age 16 years or older, (2) confirmed acute COVID-19 by positive polymerase chain reaction or rapid antigen test, (3) persistent symptoms for at least 3 months following the acute infection without a symptom-free interval, and (4) functional disability to an extent that interrupts all or most normal activities. ***Exclusion criteria*** (1) other chronic illness or demanding life situations that might explain persistent symptoms and disability; (2) sustained organ damage, such as heart and lung damage, post–intensive care syndrome, critical illness, and other severe neurologic disorders, excluding anosmia and ageusia, following acute, serious COVID-19; (3) being bedridden; and (4) insufficient command of the Norwegian language. Eligibility screening was performed by telephone contact with a research assistant.  ***Inclusion Criteria:***  **-**Patients with post-COVID fatigue as the primary symptom.  -Referral and confirmation of post-COVID fatigue by a referring physician and admitting medical specialist.  -Aged between 18 and 75 years.  -Acute COVID-19 infection at least 3 months prior to hospital admission.  -Underwent detailed anamnesis by a physician to assess symptoms.  -Provided informed consent for study participation.  ***Exclusion Criteria:***  **-**Energy level greater than 70% (assessed on a 0–100% scale) before admission.  -Lack of physician-confirmed post-COVID fatigue diagnosis.  ***Inclusion Criteria:***  **-Adults at least 3 months post-COVID-19 infection (any severity).**  **-Symptoms of brain fog.**  **-Mild to moderate cognitive impairment, defined by a Montreal Cognitive Assessment score between 10-26.**  **-Some impairment in instrumental activities of daily living, per a Cognitive Task Activity Log score ≤ 3.5.**  **-Community residents with reliable transportation.**  **-Medically stable with adequate sight and hearing to complete testing.**  ***Exclusion Criteria:***  **-Pre-existing cognitive impairment (e.g., dementia, traumatic brain injury, stroke).**  **-Severe depression or frailty.**  **A 55-year-old female with Long COVID and no improvements in fatigue over 6.5 months**    ***Inclusion Post COVID subsample:***  - SARS-CoV-2-infection and following post-COVID syndrome: Complaints that are present more than 12 weeks after the onset of SARS-CoV-2 infection and cannot be explained otherwise.  - Because of the post-COVID syndrome, at the time of the start of rehabilitation, the presence of functional limitations that may threaten the ability to work.  ***Inclusion Psychosomatic subsample:***  • There is at least on diagnosis of any mental disorder, certified by the treating physicians, e.g. panic disorder, heart-related fears, depression.  ***Inclusion Psychocardiological subsample:***  • The presence of a cardiac disease that requires rehabilitation by a specialist, e.g. specific cardiac arrhythmias, CHD, heart failure, at the same time, the presence of a mental disorder, diagnosed according to ICD-10 criteria by the clinician.  ***Exclusion:***  - Aged under 18 years  - Insufficient knowledge of the German language to fill in the  questionnaires  - patients with current psychotic symptoms, substance dependence  or abuse, and organic brain disorders,  patients in the acute phase of cardiac disease, e.g. follow-up  treatment after heart transplantation or coronary surgery during the last 6 months. |
