# Supplementary material for: Return-to-work for people living with long COVID: A scoping review of interventions and recommendations
Source: PLoS One. 2025 Oct 15;20(10):e0321891. doi: 10.1371/journal.pone.0321891 (PMC12527184; doi:10.1371/journal.pone.0321891)
Supplement: S2 Table — (DOCX) [file pone.0321891.s003.docx]

**Table S2: Categories and key messages of interventions studies for Long COVID**

| **Article** | **RTW Intervention** | **Results** | **Proportion of RTW** | **Intervention Promise** |
| --- | --- | --- | --- | --- |
| Frisk et al. 2023: A safe and effective micro-choice-based rehabilitation for patients with Long COVID:  Results from a quasi-experimental study  Altmann et al 2023. Pulmonary recovery directly after COVID-19 and in Long COVID  Brehon et al. 2022 Return-to-Work Following Occupational Rehabilitation for Long COVID: Descriptive Cohort Study  García-Molina, A. et al., (2022). Neuropsychological rehabilitation for post-COVID-19 syndrome: Results of a clinical program and six-month follow up.  Tanguay, P., et al., (2023). Post-exertional malaise may persist in Long COVID despite learning STOP-REST-PACE.  Derksen, C., et al. (2023). Longitudinal Evaluation of an Integrated Post-COVID-19/Long COVID Management Program Consisting of Digital Interventions and Personal Support: Randomized Controlled Trial.  Müller, K., et al., (2023). Impact of Rehabilitation on Physical and Neuropsychological Health of Patients Who Acquired COVID-19 in the Workplace.  Garbsch, R. et al. (2024). Sex-specific differences of cardiopulmonary fitness and pulmonary function in exercise-based rehabilitation of patients with long-term post-COVID-19 syndrome.  Frisk, B., et al. (2025). Sustained improvements in sick leave, fatigue and functional status following a concentrated micro-choice-based treatment for patients with long COVID: A 1 year prospective uncontrolled study.  Müller, K., et al. (2024). Long-term course and factors influencing work ability and return to work in post-COVID patients 12 months after inpatient rehabilitation.  Nerli, T. F., et al. (2024). Brief Outpatient Rehabilitation Program for Post-COVID-19 Condition: A Randomized Clinical Trial.  Schmid, S., et al. (2024). Effects of an integrative multimodal inpatient program on fatigue and work ability in patients with Post-COVID Syndrome-a prospective observational study.  Kupferschmitt, A., et al., (2023). First results from post-COVID inpatient rehabilitation.  Ghali, A., et al. (2023). The relevance of pacing strategies in managing symptoms of post-COVID-19 syndrome.  Oka T. (2023). A patient who recovered from post-COVID myalgic encephalomyelitis/chronic fatigue syndrome: a case report.  Hasenoehrl, T., et al., (2023). Post-COVID: effects of physical exercise on functional status and work ability in health care personnel.  Sathyamoorthy et al. 2022 Enhanced external counterpulsation for management of symptoms associated with long COVID  Wagner, B., Steiner, M., Markovic, L., & Crevenna, R. (2022). Successful application of pulsed electromagnetic fields in a patient with post-COVID-19 fatigue: a case report.  Uswatte, G., et al. (2024). Long COVID Brain Fog Treatment: Findings from a Pilot Randomized Controlled Trial of Constraint-Induced Cognitive Therapy. | **MULTIDISCIPLINARY INTERVENTIONS**  ***Micro-Choice Based Rehabilitation***  From symptom monitoring to facilitating increased physical activity and functioning through micro-choices. Using patient education, individually tailored exercise on breaking inflexible patterns, physical activity/exercise training and brief mindfulness sessions.  **Core components:**   - - Patient education   - Individually tailored exercise on breaking inflexible patterns   - Physical activity/exercise training   - Brief mindfulness sessions   **Focus shift:** From symptom monitoring to facilitating increased physical activity and functioning through micro-choices.  ***Multimodal COVID-19 rehab program***  Intervention involved educational support and group conferences, access to Long COVID ambulatory care app (MyMEDIAN@Home) and websites, detailed coaching for RTW, device-based breathing therapy and monitoring of O2 sats, medication management with Prednisolone and Colchicine, psychological counseling/meditations and psychiatric appointments as needed, and attention to social needs post-discharge.  **Program components:**   - - Educational support and group conferences.   - Access to Long COVID ambulatory care app (MyMEDIAN@Home) and websites.   - Detailed coaching for return to work.   - Device-based breathing therapy and monitoring of O2 sats.   - Medication management with Prednisolone and Colchicine.   - Psychological counseling/meditations and psychiatric appointments as needed.   - Attention to social needs post-discharge.   ***Multidisciplinary post-COVID occupational rehabilitation program***  Program involved a psychoeducational component, pacing and energy conservation guidance, breathing strategies, and tailored activity or exercise interventions  **Program components:**   - Psychoeducational - Pacing and energy conservation guidance - Breathing strategies - Tailored activity or exercise interventions   ***Multidisciplinary rehabilitation outpatient program***  **Program components:**   - Semi-structured interviews and neurocognitive battery assessments - Respiratory therapy and physiotherapy - Neuropsychological rehabilitation which included treatment for cognitive symptoms using Guttmann Neuro Personal Trainer (GNPT), emotional intervention, and training in compensatory strategies - GNPT created personalized cognitive tasks for 1-hour sessions for each person (mean = 4.1 sessions per week) - Training in compensatory strategies included: 1) promoting use of preserved cognitive resources 2) promoting the use of strategies for specific situations 3) training in the use of external aids - Emotional intervention included: 1) reduce symptoms of anxiety/depression 2) to provide tools for managing the unease associated with a disease of uncertain prognosis   ***3 neuropsychological evaluations:***   1. a pretreatment evaluation before the intervention 2. a post-treatment evaluation during the last week of the intervention, 3. a follow-up evaluation 6-7 months after the treatment.   **Multimodal:** The pretreatment and follow-up evaluations were conducted remotely via telephone, while the post-treatment evaluation was conducted either in person or remotely. Each evaluation lasted about one hour.  ***Multidisciplinary telerehabilitation program***  **Program components:**   - 14 hours of physiotherapy and occupational therapy over 12 weeks with up to 8 PT sessions and up to 6 OT sessions; on separate days - STOP-REST- PACE approach - Frequency was personalized, up to two sessions weekly - An independent coordinator assessed PEM, HRQoL, respiratory symptoms, fatigue and return to work.   ***Physical Therapy (PT) Focus:***   - Prioritize avoiding exertion that causes relapse. - Rest to conserve energy. - Pace physical and cognitive activities throughout the day. - Personalized interventions based on identified impairments. - Gentle activities (e.g., breathing exercises, stretching) for PEM-related impairments. - Progress to walking or muscle strengthening only if PEM subsided over 12 weeks. - Two experienced physiotherapists conducted sessions.   ***Occupational Therapy (OT) Focus:***   - Inspired by French-Canadian Lifestyle Redesign® for chronic pain, mild traumatic brain injury interventions, and psychological resilience concepts. - Structured course and guidebooks developed for validity and fidelity. - Five 2-hour group sessions (two 45-minute blocks with a 30-minute break) and one 60-minute individual session. - Group sessions included discussions, education, and shared experiences about Long COVID management. - Sessions ended with planning and take-home assignments. - Individual sessions focused on Personalized Engagement Plan development and follow-up. - Three experienced occupational therapists conducted sessions.   ***Therapist Training:***   - Physiotherapists and occupational therapists trained in evidence-based approaches for Long COVID, ME/CFS, and post-infectious conditions.   ***Assessment and Documentation:***   - Documented onset, severity, and frequency of PEM, well-being, fatigue, breathlessness, and other symptoms at each visit. - Medical assistance provided for events requiring immediate attention (e.g., desaturation, tachycardia, unusual breathlessness).   ***Multidisciplinary digital intervention***  **Intervention and Follow-up**:   - Initial contact: weekly telephone meetings and introduction to digital intervention platform over 2 weeks. - Unspecified treatment plan: hand hygiene and general health behaviors. - Second questionnaire (T2): symptoms, social participation, and work ability.   **Intervention Group (IG)**:   - 3-day diagnostic assessment at a specialized neurological clinic. - Post-assessment short questionnaire (T3) on symptoms. - Asynchronous digital interventions over 6 weeks tailored to assessment results. - Participated in a clinic diagnostic assessment. - Digital interventions tailored to personal capacity from assessment. - Interventions targeted low, medium, and high-capacity levels.   **Active Control Group (ACG)**:   - No clinic assessment. - Synchronous telephone contacts and asynchronous digital interventions based on main symptoms. - Biweekly meetings with personal pilots and on-demand support. - Did not undergo clinic assessment. - Digital interventions based on main symptoms (fatigue, neurocognitive, cardiorespiratory). - Personal pilots and patients chose the most fitting symptom-specific plan.   **Comparison Group (CompG):**   - Only responded to online questionnaires. - No intervention provided.   **Post-Intervention**:   - Third questionnaire (T4) on symptoms, social participation, and work ability. - Final follow-up questionnaire (T5) 6 weeks after digital interventions for both IG and ACG.   ***Core Components:***  **Support and Guidance**:   - Both IG and ACG received support from personal pilots. - Encouraged to adapt exercises individually as needed. - Exercise manual with different variants provided.   **Assessment and Customization**:   - IG assessments included cardiopulmonary exercise capacity, neuropsychological capacity, and physiological assessments. - Personal capacity categorized as low, medium, or high based on subjective and objective assessments. - Customized digital intervention plans developed accordingly.   **Digital Intervention Contents**:   - Included breathing and relaxation exercises, mindfulness training, meditation, physical strength tasks, sensory and functional training. - Focused on strengthening self-management.   **Daily Intervention Time**:   - IG: Average of 50.4 minutes (range 44.6-58.7 minutes). - ACG: Average of 41.9 minutes (range 32.5-48.7 minutes).   ***In-Patient Multidisciplinary Post-COVID Rehabilitation Program*** **Components:**  - Medical treatment and care - Comprehensive physical and psychological treatments by specialists - Complete medical, functional, motor, psychological, and cognitive measurements at four time points (at the beginning) and end of inpatient rehabilitation; 6 and 12 months after the beginning of inpatient rehabilitation. **Team Involved:** Physicians, physiotherapists, sport therapists, psychologists, social professionals, nutritionists, nurses, medical-laboratory assistants.  **Rehabilitation Components:**   - Initial illness-specific diagnostics. - Continuous adaptation of pharmacological treatments. - Psychological therapy. - Sports therapy. - Physiotherapy. - Respiratory therapy. - Health promotion interventions (e.g., smoking cessation, stress management, physical activity).   **Psychological Therapy:**   - Initial psychological interviews. - Participation in education groups: “Anxiety”, “Depression”, “Sleep & Stress”, “Cognitive Training”. - Cognitive training using “Fresh Minder 2, 3, and 4” programs. - Weekly group meetings for mindfulness, art therapy, and post-COVID-19 group therapy. - Individual psychological and psychiatric sessions as needed.   **Sports and Physiotherapy:**   - Individual training programs based on initial physical assessment. - ***Endurance Training:*** Nordic walking, ergometer training. - ***Strength Training:*** Medical training therapy, stair climbing. - ***Coordination and Mobility:*** Coordination, mobility, and fascia training. - ***Relaxation Exercises:*** Progressive muscle relaxation.   **Respiratory Physiotherapy:**   - Mobility and respiratory techniques. - Inspiratory muscle training. - Specific manual therapy. - Focus on reducing breathing resistance, managing breathlessness, and training respiratory muscles.   ***In-Patient Multidisciplinary Rehabilitation Program with individualized medical rehabilitation***  At enrollment a full clinical assessment including symptom limited cardiopulmonary exercise testing (CPET) and pulmonary function (spirometry) tests were performed. Valid questionnaires were applied at enrollment, discharge, and 6 months follow-up to assess disease perception and workability.  **Questionnaires administered evaluated:**   - fatigue - quality of life - anxiety - depression - work ability   A 4-week inpatient rehabilitation program which included active, cognitive, and passive therapies. Active therapies were completed at a mean of 12 sessions per week, cognitive therapies average about 8 sessions per week and passive therapies were about 12 times per week. Interventions were tailored based on patient performance and adjusted by therapists throughout rehabilitation process  **Active therapies involved:**   - physical rehabilitation (strength, endurance, and respiratory training, including group exercise, aerobic ergometer, aqua fitness, walking and circuit training) - inspiratory muscle training   **Cognitive therapies involved:**   - disease education - psychological counseling - nutrition education - stress management - coping strategies   **Passive therapies included:**   - relaxation techniques, such as, muscle relaxation, heat therapy, and massages   ***Micro-Choice Based Rehabilitation (see above original study)***  From symptom monitoring to facilitating increased physical activity and functioning through micro-choices. Using patient education, individually tailored exercise on breaking inflexible patterns, physical activity/exercise training and brief mindfulness sessions. 1 year follow-up.  **Core components:**   - - Patient education   - Individually tailored exercise on breaking inflexible patterns   - Physical activity/exercise training   - Brief mindfulness sessions   **Focus shift:** From symptom monitoring to facilitating increased physical activity and functioning through micro-choices.  ***In- Patient Multidisciplinary Post-COVID Rehabilitation Program. (see above original study)*** **Components:**  - Medical treatment and care - Comprehensive physical and psychological treatments by specialists - Complete medical, functional, motor, psychological, and cognitive measurements at four time points (at the beginning (T1) and end (T2) of inpatient rehabilitation; 6 (T3) and 12 (T4) months after the beginning of inpatient rehabilitation. **Team Involved:** Physicians, physiotherapists, sport therapists, psychologists, social professionals, nutritionists, nurses, medical-laboratory assistants.  **Rehabilitation Components:**   - Initial illness-specific diagnostics. - Continuous adaptation of pharmacological treatments. - Psychological therapy. - Sports therapy. - Physiotherapy. - Respiratory therapy. - Health promotion interventions (e.g., smoking cessation, stress management, physical activity).   **Psychological Therapy:**   - Initial psychological interviews. - Participation in education groups: “Anxiety”, “Depression”, “Sleep & Stress”, “Cognitive Training”. - Cognitive training using “Fresh Minder 2, 3, and 4” programs. - Weekly group meetings for mindfulness, art therapy, and post-COVID-19 group therapy. - Individual psychological and psychiatric sessions as needed.   **Sports and Physiotherapy:**   - Individual training programs based on initial physical assessment. - ***Endurance Training:*** Nordic walking, ergometer training. - ***Strength Training:*** Medical training therapy, stair climbing. - ***Coordination and Mobility:*** Coordination, mobility, and fascia training. - ***Relaxation Exercises:*** Progressive muscle relaxation.   **Respiratory Physiotherapy:**   - Mobility and respiratory techniques. - Inspiratory muscle training. - Specific manual therapy. - Focus on reducing breathing resistance, managing breathlessness, and training respiratory muscles.   ***Brief Outpatient Rehabilitation Program***  The intervention consisted of 2 to 8 outpatient sessions over a period of 2 to 6 weeks, following the study site’s standard clinical approach without additional resources. It was theoretically based on the Cognitive Activation Theory of Stress (CATS), which explains how sustained stress responses can contribute to persistent symptoms. The goal was to modify cognitive expectancies that influence symptom duration and severity.  The rehabilitation program had two stages:   1. **Clinical Assessment (Stage 1):** A physician conducted a medical evaluation to rule out differential diagnoses, validate symptoms, and provide psychoeducation based on CATS. This included explaining normal stress responses and how infections like COVID-19 could trigger maladaptive reactions, reinforcing that symptoms were temporary and modifiable. 2. **Cognitive Behavioral Therapy (Stage 2):** CBT-trained physiotherapists guided patients using nondirective communication, Socratic dialogue, and guided discovery. Sessions focused on modifying symptom-related expectancies, encouraging engagement in physical and mental activities, and discouraging excessive symptom monitoring. Patients were supported in exploring new activities to facilitate recovery   ***Multimodal Inpatient Rehabilitation***  Patients hospitalized for post-COVID syndrome received a 14-day multimodal integrative treatment tailored to individual needs.  The **intervention included** a wide range of therapies, such as:   - Vitamins/Nutrients - Hydrotherapy & Thermotherapy - Mind–Body Medicine - Phytotherapy (herbal treatments) - Exercise Therapy - Naturopathy - Nutrition Therapy - Other personalized therapeutic approaches   Treatment was customized based on patient-specific symptoms and rehabilitation goals.  ***Multimodal Inpatient Rehabilitation***  Measures at intake and discharge:   - Beck Depression Inventory II (BDI-II) - The Hamburg module for the assessment of psychosocial health in clinical practice (HEALTH-49) - Work-related/socio-medical parameters - 6 Minute Walk Test (6MWT)   **Intervention: program components:**   - **Individual and group psychotherapy (CBT)** - **Individualized aerobic exercise training** - **Body awareness training and breathing therapy** - **Relaxation techniques and cognitive training** - **Social counseling**   **SINGLE INTERVENTIONS**  ***Physician-lead intervention.*** included patients meeting the World Health Organization definition of Post-COVID-19 Syndrome (PCS) who attended the Internal Medicine Department of Angers University Hospital, France, between June 2020 and June 2022, with follow-up until December 2022.  **Pacing program components:**  - Involves adjusting **physical, cognitive, and emotional activities** to align with the individual's illness-imposed limits  - Closely related to the **energy envelope theory,** which emphasizes staying within a sustainable activity level to prevent both **overexertion**(which can trigger symptom exacerbation and post-exertional malaise) and **under exertion**, ensuring gradual functional improvement.  **Assessments:** self-reports on health status (1-5 scale), fatigue levels (Fatigue Severity Scale), persistent recurrent or new symptoms, impact of LC on patient’s activities (especially occupations), and degree of adherence to pacing strategies (Engagement in Pacing Subscale of the Activity Pacing and Risk of Overactivity Questionnaire).  **Pacing Strategy Objective:** Prevent exacerbation of symptoms, particularly fatigue and post-exertional malaise (PEM), while maintaining activity.  **Three Pillars of Pacing Strategies:**   1. **Energy Envelope:**  - Identify current physical and mental capacity limits in daily activities. - Do not exceed these limits. - Prioritize activities, combine periods of activity with rest, and split/switch activities.  1. **Prevent Worsening/Relapse:**  - Identify PEM triggers such as physical, mental, emotional stressors, orthostatic intolerance, hormonal factors, environmental factors, sensory stimuli, certain foods, and infections. - Recognize new symptoms that precede PEM (e.g., mood disorders, nausea, headaches, vertigo, dyspnea, tingling/burning sensations) as warning signals.  1. **Gradual Activity Increase:**  - Increase activities cautiously and progressively only when symptoms are stabilized.   **Patient Education:**   - Received a leaflet explaining the basics of pacing and implementation tips.   **Diary Keeping:**   - Record physical and mental capacity limits, fatigue triggers, other baseline symptoms, and possible PEM warning signals.   ***Physician-Led Intervention.*** The patient was a previously healthy 55-year-old woman who worked as a nurse and became ill with COVID-19 pneumonia. She then presented with severe fatigue, post-exertional malaise, dyspnea, pain, cognitive dysfunction, tachycardia, and exacerbation of fatigue on physical exertion, which persisted for more than 6 months after her recovery from COVID-19 pneumonia. She was bedridden for more than half of each day.  **Program Components.** Used the following treatment approaches:   - **Eating Habits and Supplements:** Provided instructions on diet and supplementation. - **Cognitive and Behavioral Modifications**: Guidance on coping with physical, emotional, and cognitive fatigue. - **Conditioning Exercises**: Exercises to address deconditioning from fatigue and dyspnea. - **Pharmacotherapy**:   - Amitriptyline.  - Hochuekkito, a Japanese herbal (Kampo) medicine.  ***Exercise intervention***  Effects of exercise on symptoms of health care workers with Long Covid.  **Duration:** 8 weeks  **Intervention components:**  - Two supervised resistance exercise sessions per week  - Individual aerobic exercise recommendations  - Measurements at baseline, at 4 weeks, and at end of program (8 weeks)  ***Resistance program:***  **Design**: Circuit training with body weight and resistance bands.  **Exercises**:   - - Squats   - Glute bridge   - Hip abductor walks   - 45° standing back extension   - Push-ups   - Low row   - Planking   - Shoulder external rotation   **Difficulty Levels**: Varied to match participants' fitness levels.  **Structure**:   - - Warm-up routine followed by two sessions of eight exercises.   - Initial phase (Weeks 1-2): 30 seconds exercise, 30 seconds rest.   - Progression: Increase by 10 seconds every two weeks.   - Final phase (Weeks 7-8): 60 seconds exercise, 60 seconds rest.   **Intensity**:   - - Weeks 1-2: Submaximal (RPE 7-8 on a 10-point scale).   - Weeks 3-8: Higher intensity (RPE 9-10).   **Aerobic Exercise**:   - **Equipment**: Activity tracker watch (Garmin Venu SQ). - **Guidelines**:   - Perform as much aerobic exercise as feasible.   - Minimum: Three sessions of 20 minutes of moderate aerobic exercise per week.   - Focus on low intensities, primarily at ventilatory threshold 1 (VT1).   **ENHANCED EXTERNAL COUNTERPULSATION (EECP)**  ***Enhanced External Counterpulsation (EECP) for Long COVID.***  Intervention included EECP for 1-hour sessions for 35 sessions or a modified regimen of 15 sessions.  **Program components:**   - EECP: 1-hour sessions for 35 sessions or a modified regimen of 15 sessions.   **ELECTROMAGNETIC FIELD THERAPY**  ***Electromagnetic Field Therapy***  The intervention involved pulsed electromagnetic field (PEMF) therapy using the **Papimi™ electromagnetic field therapy device** (class IIa certified).  **Duration:** **10 sessions over 5 weeks** (twice weekly), with each **30-minute session**targeting specific body areas based on the manufacturer’s guidelines  **Treatment Protocol:**  **Positions & Timing:** Supine position with targeted application:   - **Epigastric/abdominal area** – 6 min - **Sternum** – 3 min - **Dorsal area (lungs & adrenal region)** – 6 min - **Pelvic floor** – 6 min - **Soles of feet** – 6 min - **Pulse Rate:** 2.5 Hz for the dorsal area, 1 Hz for all other locations.   **Treatment Intensity:** Adjusted based on tolerance, using an applicator spool distance of 0–4 cm.  **Equipment Adjustments:** Started with an 18 cm spool, later switching to a 20 cm spool for all areas except the pelvic floor.  **Power Settings:** 75% for abdominal, sternal, and pelvic floor areas; 100% for dorsal and plantar applications.  ***Contraindications*** (electronic implants, pregnancy, and metal rings in the body) were ruled out before treatment.  **CONSTRAINT-INDUCED COGNITIVE THERAPY**  **Intervention:** The study enrolled **16 community-dwelling individuals** three months post-COVID-19 with **mild cognitive impairment and IADL dysfunction.** Participants were **randomized** to either **Immediate Constraint-Induced Cognitive Therapy (CICT) or treatment-as-usual (TAU)** with a later crossover to CICT.  **CICT components:**   - **Modified Behavior Change Techniques from Constraint-Induced Movement Therapy** - **Speed of Processing Training using a computerized cognitive-training program**   **Feasibility Criteria:**   - **≥80% completion rate** - **High satisfaction and at most moderate difficulty** - **Fewer than two serious adverse events**   **Primary Outcome Measures:**   - **IADL performance**(Canadian Occupational Performance Measure) - **Employment status** - **mBrain fog** (Mental Clutter Scale)   **Testing time:** Immediate Constraint-Induced Cognitive Therapy (CICT): Before and after treatment. Treatment-as-usual (TAU): Baseline 1 & 2 (during TAU) and before/after crossover to CICT.  **CICT Intervention:**  Consisted of **36 hours of training** over **2 to 7 weeks,** tailored to participants’ needs.  **Each session included:**   - **Speed of Processing Training (SOPT) (20%)** – A computerized program requiring participants to rapidly detect, identify, and locate visual targets with increasing difficulty. - **In-Lab Instrumental Activities of Daily Living (IADL) Training (35%)** – Task-based training using **shaping principles** (small, structured steps with reinforcement) and **task practice** (continuous, real-world tasks like making a shopping list or scheduling appointments). - **Transfer Package (30%)** – A cognitive adaptation of the **Motor Transfer Package,** helping participants apply learned skills to real-world settings. - **Rest Periods (15%)** – Incorporated to optimize cognitive performance and reduce fatigue.   The intervention aimed to enhance **cognitive function and IADL performance,** supporting participants in regaining independence. | - Work and Social Adjustment Scale mean score from baseline at 21.9 reduced to 6.9 at 3-month (larger than MCID)  The rehabilitation program led to significant pulmonary recovery and improved outcomes in both groups, facilitating return to work for most patients. Dysfunctional breathing patterns and diaphragm dysfunction are common in both acute and Long COVID cases, requiring early diagnosis and targeted therapy. Specialized rehabilitation programs are effective in managing these respiratory impairments and supporting recovery.  Outcomes would likely improve with increased availability of modified duties and timelier rehabilitation  The results obtained in this study suggest that, regardless of the underlying condition, cognitive rehabilitation is a useful tool for treating alterations affecting cognitive function. neuropsychological rehabilitation may be less effective in older adults due to the ageing process (whether normal or pathological).  Return to work remained a significant challenge for participants. Results show that despite the integration of pacing, most patients were unable to regain work capacity.  - The regression analysis revealed that a greater reduction in symptoms does not predict a higher reported work ability when controlling for age, sex, the BMI, and previous health conditions    Significant improvements in physical performance and neuropsychological health outcomes were determined. Moreover, healthcare workers showed a significantly greater reduction in depressive symptoms compared to non-healthcare workers. Most participants were still suffering from the impact of COVID-19 at rehabilitation discharge thus, ongoing strategies in aftercare are necessary to improve their work ability.  - significant improvements in physical fitness  - female patients showed greater improvements in workload, oxygen uptake and respiratory minute ventilation  - males had a lower baseline cardiopulmonary exercise capacity  - significant improvements in fatigue, quality of life, and mental health (depression and anxiety) – with females showing greater improvement than males  The functional levels (Visual Analogue Scale) increased from 52.9 at baseline to 68.7 at 3-month follow-up to 75.9 at 1 year follow-up. The Work and Social Adjustment Scale scores improved with an estimated reduction in mean scores of 6.9 and 9.7 (clinically significant).  Fatigue was identified as the main predictor of reduced work ability and RTW, with each unit increase in fatigue severity decreasing the odds of RTW by 3.1%. In addition, physical capacity and subjective health status were significant predictors of perceived work ability.    In this Randomized clinical trial, a brief outpatient rehabilitation program with a cognitive and behavioral approach in patients with PCC was effective and safe  **-**The results indicate a substantial and sustained increase in work capacity following the multimodal rehabilitation program.  The results of this study show that rehabilitation patients with post-COVID-syndrome have a significantly elevated, but lower psychological burden than patients in psychosomatic or psychocardiological rehabilitation. Post-COVID appears to be a multifactorial clinical picture that includes somatic and psychological components and has socio-medical and social implications  - High recovery and improvement rates were observed in post-COVID syndrome (PCS) patients following the implementation of pacing strategies  - Greater adherence to pacing strategies led to faster recovery, improved health status, and increased return-to-work rates.  - Findings support the use of pacing for PCS management, like ME/CFS, emphasizing the need for physician awareness and patient education programs.  The patient made a complete recovery and returned to work as a nurse.  - Physical exercising showed to be an effective intervention method in the rehabilitation of COVID-19 survivors suffering from post-COVID syndrome by positively affecting both physical and mental health.  - In health care workers suffering from post-COVID syndrome, increases in physical performance are directly related to improvements in work ability.  - The 30 s sit-to-stand test (30secSTS) showed promising results as clinical assessment tool.  - The results of this study indicate that physical exercising will need to play a large and substantial role over the next years in the rehabilitation of COVID-19 survivors suffering from post-COVID-19-syn- drome as it positively affects both physical and mental dimensions of the post-COVID-19-syndrome as well as work ability.  - Initial trial utilized 15 sessions as proof-of-concept.  - 25 to 35 sessions may ultimately be the most suitable.  - Improvement in brain fog symptoms observed in all patients.  The use of pulsed electromagnetic field therapy with a device that allows sufficient penetration of the body tissue might be a promising physical modality to manage post-COVID-19 fatigue syndrome, which could reduce clinical and economic health consequences  CICT has promise for reducing brain fog, improving IADL, and promoting returning-to-work in adults with Long COVID. CICT produced improvements in both everyday function and psychological distress | - Reduction in sick leave from 63% to 43% at 3-month follow-up  - overall mean degree of sick leave reduced from 51% to 30%  - Before discharge only 5% of participants with acute COVID-19 were rated as fit for work immediately compared to 33% of participants with Long COVID  - At 6 months most participants with acute COVID-19 were able to RTW vs only 33% of participants with Long COVID  - Overall, only about 5% of participants with COVID-19 were unfit for work vs 29% of participants with Long COVID  - Only 43 (53%) individuals returned to work at program discharge  - 40 (93%) of those who returned to work returned to modified duties  - 33.3% returned to work  - 44.9% were temporarily unable to work  - 77.3% of those who had returned to work described difficulties performing workplace activities  - In the study group, 34.5% (n = 19) were working and 43.6% (n = 24) were temporarily unable to work  - In the control group, 28.6% (n = 4) were working and 50% (n = 7) were temporarily unable to work.  - 25% returned to work, 75% continued to be on medical leave after 12 weeks telerehabilitation  - In 12 weeks, 15/20 (75%) were unable to RTW and remained on Medical Leave  - In week 12, 3/20 (15%) returned to work at part-time and 2/20 (10%) returned to work at full-time  - For work ability, there was no significant difference between T2 and T4, but the work ability was higher in the IG and ACG compared to the CompG  -Participants reported poor work ability, and 72.5% of them were still unable to work after discharge from rehabilitation  - The total score of the WAI between T1 (24.75, IQR: 21–28) and T2 (24.75, IQR: 21–28) did not increase significantly (p = 0.408)  - at 6 months, workability showed a significant improvement (*p*=0.003) compared to admission with no significant gender differences  After 1 year follow-up sick leave was reduced. Sick leave at baseline was 65% which reduced to 43% at 3 month follow up to 23% at 1 year follow-up.  - At T4, the median of WAI total score indicated poor work ability, which significantly worsened over time (p < 0.001; r = 0.484)  - At T4 (12 month follow up), 54 (47%) post-COVID patients had successfully returned to work, while 57 (50%) remained unable to do so. Among those not working, 6 (5%) were in the process of reintegration, 8 (7%) were receiving a disability pension, and 3 (3%) were on an old-age pension.  At T1 (completion of study) from the usual group 41 (39%) returned to work and from the intervention group 73 (58%) returned to work. At T2 (6-month follow-up), 29 (28%) usual group returned to work and 58 (46%) of intervention group returned to work.  Significant RTW self-efficacy at T1 (p=0.01).  **-**Self-reported work ability (on an 11-point scale) significantly improved from 2.5 before treatment to 4.3 immediately after the 14-day inpatient stay.  This improvement remained stable six months later, with a mean score of 4.4.  - Psychosomatic symptoms increased from 28.6% at intake to 30.6% at discharge  - Post COVID symptoms decreased from 41.0% at intake to 35.3% at discharge  - Psychocardioological symptoms decreased from 42.0% at intake to 32.0% at discharge  - Of the 86 patients, 29 (33.7%) recovered and returned to work  - Of these, 10/29 (34.5%) returned to full-time work and the rest 19/29 (65.5%) returned to work on a part-time basis  - A relatively high recovery/improvement rate (49/86 patients, 57%) after pacing implementation  Complete recovery and returned to work as a nurse (n=1)  Post-COVID exercise intervention improved physical fitness, psychological outcomes and workability in healthcare workers (HCWs)  - Ability to return to work assessed qualitatively  - All 6 patients (100%) unable to work previously returned to work/school after EECP therapy  Workability improved from critical (21.5 points) before treatment to good (40 points) after the intervention.  - Significant advantage for Immediate-CICT over TAU (*p* = 0.048).  - 80% of Immediate-CICT participants who had not retired prior to COVID-19 onset resumed a full set of work duties after treatment; none did so after TAU  -Immediate-CICT Group:  -After Immediate-CICT, 4 of 5 participants resumed full-duty work.  TAU Group:  -None were able to return to work before or after TAU.  -Retirement: 2 participants in each group had retired before COVID-19 onset. | Somewhat promising  Not promising  Promising  Somewhat promising  Somewhat promising  Not promising  Somewhat promising  Promising  Somewhat promising  Somewhat promising  Promising  Promising  Not promising  Somewhat promising  Promising  Uncertain  Promising  Uncertain  Promising |
