# Supplementary material for: Return-to-work for people living with long COVID: A scoping review of interventions and recommendations
Source: PLoS One. 2025 Oct 15;20(10):e0321891. doi: 10.1371/journal.pone.0321891 (PMC12527184; doi:10.1371/journal.pone.0321891)
Supplement: S3 Table — (DOCX) [file pone.0321891.s004.docx]

**Table S3. Recommendations for Return-to-Work From Guidelines**

| **Guidelines title** | **Year published** | **Country** | **Purpose** | **Guidelines for** | **Health Care Professionals involved** | **Recommendations for RTW** |
| --- | --- | --- | --- | --- | --- | --- |
| Society of Occupational Medicine – *Long COVID and Return to work: what works?*  Society of Medicine issues Long Covid and COVID return-to-work guidance.  *COVID-19 RTW guide – for recovering workers*  Canadian Thoracic Society position statement on rehabilitation for COVID-19 and implications for pulmonary rehabilitation  COVID-19 return to work guide for managers  Supporting patients with Long COVID return to work.  Experiences of workers with post-COVID-19 symptoms can signpost suitable workplace accommodations  COVID-19 infection and Long COVID – guide for managers    Recommendations for the recognition, diagnosis, and management of Long COVID: a Delphi study  In the wake of the pandemic: preparing for Long COVID  Managing the long-term effects of COVID-19: Summary of NICE, SIGN, and RCGP rapid guideline    The Importance of Keeping Patients with Post-Acute Sequelae of SARS-CoV-2 Infection (Long COVID) Engaged in Work. | 2022  2021  2021  2021  2021  2022  2021  2021  2021    2021  2021 | UK  United Kingdom  Canada  UK  UK  UK  Europe  UK  Europe, World Health Organization  London, UK  USA | To provide guidance on the identification and management of Long COVID – particularly regarding return to work.  To address the different approaches to the management of Long COVID across the UK and elsewhere.    To offer guidance for workers from Occupational Health Professionals on how to manage getting back to work after COVID-19 infection and Long COVID.  -To provide rapid guidance for pulmonary rehabilitation in adults with COVID-19 illness.  To offer guidance from Occupational Health Professionals on how managers can support workers to get back to work with ongoing symptoms following COVID-19 infection and Long COVID.  To present the guiding principles of supporting people with Long COVID to return to work based on a review of the current literature and guidance from the Faculty of Occupational Medicine of the Royal College of Physicians  An exploratory survey was undertaken to provide empirical evidence of the work-relevant experiences of workers recovering from COVID-19.  Information in this guide to help support workers returning to work following a COVID- 19 infection or Long COVID for managers/ employers.    To provide a rapid expert guide for GPs and Long COVID clinical services.  This policy brief seeks to raise awareness of Long COVID and to provide a resource for those in decision-making roles, setting out in basic terms what we know about this condition and what the policy options are for developing a response that leaves no one behind.  The rapid guide covers care for people with signs and symptoms that continue for more than four weeks, and which developed during or after an infection consistent with COVID-19, and which are not explained by alternative diagnoses.  A letter to the editor discussing the importance of work engagement for patient with Long COVID | It will be of use to occupational health providers, employers, workers, people with Long COVID, HR personnel, managers, medical, allied health professionals and unions.  For Long COVID recovering workers; anyone already working, looking for work, or wanting to return to work  Healthcare professionals and multidisciplinary rehabilitation teams  Managers/ supervisors  Long COVID patients and health care professionals  All members involved in care continuum of patients with Long COVID (i.e. patient, health care professionals, insurers, etc.)  Managers/ supervisors  General Practitioners/doctors and Long COVID clinical services  Decision-maker roles  Clinicians    Physicians | -Occupational therapy  -Physiotherapy/ respiratory physiotherapy  -Speech and language therapy  -Neuropsychologist  - Psychologist  Occupational therapy  Physiotherapy  counseling (have to ask for request for these)  - various health care professionals and seek input from occupational therapy and psychology as needed  Occupational health and/or psychological or wellbeing services  N/A  “Health care professionals”  Occupational health and/or psychological or wellbeing services  Multiple health care  professionals  Multi- disciplinary approach to treatment  Holistic multi- disciplinary  Physician-lead | **1. Early intervention, integration, and rehabilitation for early recovery and return to work**  **2. Support of employers, the line manager/supervisor, and human resources**  - HR professionals should collaborate with occupational health, line managers, and employees to review job roles and determine individual support and adjustments  - research states a whole system approach is needed to support RTW  - IGLOO framework: emphasizes prolonged phased RTW over many months  - PIES principles for supervisors: Proximity (active supportive management), Immediacy (‘Nip it in the bud’ approach), Expectancy (Communication and anticipation of recovery, early professional help if needed), Simplicity (Use of brief, uncomplicated interventions like problem solving)  - need flexible approach to support fluctuating and unpredictable symptoms of LC  - policies should support employees in managing their health and practicing self-care  **3. Early planning for RTW participation**  - optimal approach 🡪 evidence-informed early stepped-care based on biopsychosocial principles  - bio component – manage symptoms and obtain support for persistent symptoms  Psychological aspect – address uncertainties and misinformation, provide accurate information to reduce fear and uncertainty, avoid “can’t do” and boom-bust cycle and promote pacing and “can do”  -RTW considerations: address topic of RTW supportively and ASAP, focus on relevant symptoms and job modifications  -flexible, regularly reviewed, long-term RTW planning involving workers, line managers, OH professionals, HR, and supportive organizational culture  ***Key aspects of RTW plan:***   - Focus on what can be done, not what cannot - Ensure all parties are supportive - Identify obstacles to RTW using biopsychosocial framework - Develop actions to overcome obstacles (temporary job modifications, pacing, time off for treatment, etc.) - Ensure healthcare is work focused - Negotiate and agree on actions with line manager and health professionals - Set RTW date and timeline for actions - Agree on review schedule for the plan   - Emphasize RTW achievable with appropriate support  - ongoing discussion with all individuals involved is critical for effectiveness of adjustments and support  ***Work & Health***   - Significant workplace exertion contraindicated in patients with uninvestigated chest pain due to high risk of exercise-induced sudden cardiac death in acute myocarditis and common microvascular angina - Consider persisting low-grade cardiac injury in protracted C19 illness, especially for jobs involving strenuous physical activity - Cardiorespiratory clearance required after infection before resuming strenuous workplace exertion - Myocarditis requires three to six months and an “all-clear” from a cardiologist before returning to heavy exertion - Cognitive dysfunction is common but may not become apparent until RTW - Cognitive assessment needed for safety-critical tasks - Orthostatic intolerance for autonomic dysfunction is almost universal in LC   **1. Returning to work**  - ***health disclosures and support*** 🡪 optional to disclose health problems but recommended for support and possible referral to Occupational Health (OH) for Long COVID  ***- health assessments for special roles*** 🡪 legally advised to inform OH of health conditions for specific jobs with special health and safety standards  ***- RTW meeting/interview*** 🡪 manager should hold a meeting before RTW to discuss support needs, arrange a review soon after RTW, review workloads to avoid excessive pressure, and involve Human Resource partners or union representatives if helpful  - ***Medical clearance before RTW*** 🡪 necessary for jobs involving heavy lifting or exertion (heart and lung checks required), additional health checks for safety-critical roles, review previous work restrictions if pe-existing conditions aggravated by COVID-19, and discuss adjustments to work duties with managers  - suggestions/advice 🡪 make suggestions based on your experience and health condition, seek advice from doctors on work-related activities, consult an occupational physician or advisor if uncertain, and discuss reasonable adjustments with your manager (consider permanent changes if needed later)  **2. some examples of adjustments to work duties**  ***- phased return*** 🡪 Graduated RTW (adaptable as needed), longer phased return often required for Long COVID (exceeding the average 4 weeks), risk of symptom relapse from overexertion (monitor symptoms closely), and flexible adjustments best worked out between employee and manager  ***- Adjustment examples:***   - Timing alterations (changes to start and finish times and breaks) - Hours adjustments (shorter workdays or days off between workdays) - Workload changes (fewer tasks within a given time, more time for usual tasks) - Work patterns (regular breaks) - Duties/tasks (temporary changes to these) - Support mechanisms (clear supervision/buddy system/time off for appointments/avoid working in isolation) - Clear objectives and review mechanisms - Part-time working from home or remote options - Equipment adjustments 🡪 blue light screen filters, voice-activated software, ergonomic office chairs, enhanced moving/handling equipment - Other 🡪 legal requirement for reasonable adjustments if condition considered disability (UK ACT)   **3. The Employer’s overall responsibilities**  • General policies to ensure ‘good work’ for all  • A sickness absence policy  • Flexible working policies  • Health and Safety at Work obligations  • Disability and other Equality policies  - Pulmonary rehabilitation can help with symptoms that are impacting RTW  - ***Adaptations for pulmonary rehabilitation for patients with Long COVID to aide with RTW include:***   - Begin aerobic exercise at lower intensities - Conservative progression and symptom monitoring to prevent post-exertional malaise - Gradual introduction of strengthening exercises - Modify education modules for Covid-19 specific challenges   ***- Considerations for patients with Long COVID:***   - Activity-induced mental and cognitive fatigue - Sleep and mood disturbances - Persistent cognitive problems - Difficulty with RTW   - Seek occupational therapy and psychology input if needed for additional challenges  - Recommended outcome measures can include RTW items as applicable  ***- Targeted and individualized pulmonary rehabilitation:***   - Ensure accessibility for marginalized groups - Use accessible language - Provide access to transportation and virtual rehab options   - Manager’s role is vital as they are often first point of contact, and they are able to put in place job modifications or work adjustments to cope with work and their healing upon return  Key steps for managers to follow when supporting worker with RTW:  ***Step 1: stay in touch while the worker is absent from work***   - Maintain contact - Fit note (doctor’s note) - Agree what to tell others (confidentiality) - Access to occupational health/wellbeing services - Give them permission to rest and recuperate   ***Step 2: prepare for worker’s return***   - Some people require medical clearance before RTW - Put yourself in their shoes – see what you can do - Arrange a RTW conversation to agree to a RTW plan - Consider using Access to Work (government organization in UK)   ***Step 3: hold a RTW conversation***   - Think about work modifications and workable options - Involve important partners (HR, union reps, etc.) - Tell them to seek medical advice as needed - Check in with them and set them at ease - Explore solutions to their concerns/barriers - Talk about work schedule, work priorities, job modifications, monitoring, progression, and agree on a RTW plan - Flexibility is necessary - Share plan with occupational health and HR or possible work buddies   ***Step 4: Provide support during the early days of the RTW***   - Make sure you are available to welcome them back day 1 - Give them permission to take things slowly upon return - Remind them of the work priorities, schedule, and modifications you have agreed upon - Update them on any new changes that have been made - Arrange regular check-ins   ***Step 5: Provide ongoing support and review regularly***   - Communicate regularly and openly - Review workloads - Seek advice from HR where appropriate   -If unsure about organisation’s return to work processes providing working to support returning worker, ask Human Resources professional for advice  -Understand/review policies/procedures and human resources involvement  ***Examples of job modifications***   - Phased return and working hours – longer phased returns for LC - Alterations to the timing of work (start, finish, breaks, etc.) - Alterations of hours worked (i.e. shorter days, days off between workdays, etc.) - Alterations to shift work (i.e. consider eliminating night shift to have them work at best hours) - Alterations to the patterns of working (i.e. pacing, regular and/or additional breaks) - Alterations to workload (i.e. fewer tasks than normal within a time, more time to complete usual tasks and not to work tight deadlines) - Temporary changes to duties/tasks (i.e. alternative work/duties) - Support – someone to ask or check with, “buddy system,” time off for appointments, not working in isolation - Clear objectives and review mechanisms - Working from home part of the time where possible - Regular checks on whether the symptoms are fluctuating - Seek advice from OH/HR – especially if is likely considered a disability   Guiding principles for supporting people with Long COVID to RTW (based on current literature):  Examination:   - Include the one-minute sit-to-stand test - Include the three-minute active stand test   Sleep Hygiene:   - Essential, especially if napping frequently during the day - Assess sleep history   Mental Health Considerations:   - Symptoms of depression and anxiety common in LC patients - Poor concentration and fatigue can impact functional impairment especially at work - Consider referral to psychological services   Occupational history:   - Assess patient’s job, daily tasks, working hours, shift work, physical demands, and safety-critical aspects of job (i.e. driving, operating machinery, emergency services, etc.)   Communication with employer:   - Contact line manager to determine necessary adjustments for RTW (i.e. gradual RTW)   Tailored RTW plan:   - Adapt the RTW plan to patient’s symptoms - Reassure patients that a mild increase in symptoms on RTW is unlikely to worsen their condition   **RTW accommodations (section 4.3):**  ***Workplace support***   - Manager support - Face-to-face support - Regular check-ins   ***Accommodating workplace:***   - Changes to workload - Pacing tasks - Rest breaks - Reduced hours   ***Modifying policies***   - Sick leave - COVID-19 transparency - Inclusion of bullying/harassment policies   ***Flexible and collaborative RTW planning:***   - Autonomy over hours - Graded approach   ***COVID-19 awareness and compassion programmes:***   - Training on COVID-19 awareness - Myth busting - Differentiating fatigue vs. feeling tired at work   ***Employer benefits:***   - More support leads to quicker recovery and greater productivity   ***Key elements for RTW planning:***   - Flexible and positive planning - Recognition that goals may need adjustment due to symptom resurfacing - Realistic expectations of worker roles - Frequent review of RTW plans   ***Work modifications:***   - More breaks - Allow more time for tasks - Pacing tasks - Self-management of job requirements - Defer complex responsibilities until adjustment   ***Improving health and work outcomes:***   - Scope for workers to self-manage symptoms alongside job demands - Focus on work-relevant symptoms to avoid counterproductivity   ***Framework:***   - Biopsychosocial   - Manager’s role is vital as they are often first point of contact, and they are able to put in place job modifications or work adjustments to cope with work and their healing upon return  Key steps for managers to follow when supporting worker with RTW:  ***Step 1: stay in touch while the worker is absent from work***   - Maintain contact - Discuss legal rights and duties - Agree what to tell others (confidentiality) - Access to occupational health/wellbeing services - Give them permission to rest and recuperate - Provide information to workers on any rehabilitation policies   ***Step 2: prepare for worker’s return***   - Some people require medical clearance before RTW - Put yourself in their shoes – see what you can do - Arrange a RTW conversation to agree to a RTW plan - Consider using Access to Work (government organization in UK) - Consult occupational health or physician on how to adapt workplace - Seek advice/support schemes using government provisions – generic or COVID-19 specific   ***Step 3: hold a RTW conversation***   - Think about work modifications and workable options (i.e. cognitive, physical and emotional) - Involve important partners (HR, union reps, etc.) - Tell them to seek medical advice as needed - Check in with them and set them at ease - Explore solutions to their concerns/barriers - Talk about work schedule, work priorities, job modifications, monitoring, progression, and agree on a RTW plan - Flexibility is necessary - Share plan with occupational health and HR or possible work buddies - Avoid any unsolicited adjustments   ***Step 4: Provide support during the early days of the RTW***   - Make sure you are available to welcome them back day 1 - Give them permission to take things slowly upon return - Remind them of the work priorities, schedule, and modifications you have agreed upon - Update them on any new changes that have been made - Arrange regular check-ins   ***Step 5: Provide ongoing support and review regularly***   - Communicate regularly and openly - Review workloads - Seek advice from HR where appropriate - Keep any eye on the workload of other workers   ***Examples of job modifications***   - Phased return and working hours – longer phased returns for Long COVID - Alterations to the timing of work (start, finish, breaks, etc.) - Alterations of hours worked (i.e. shorter days, days off between workdays, etc.) - Alterations to shift work (i.e. consider eliminating night shift to have them work at best hours) - Alterations to the patterns of working (i.e. pacing, regular and/or additional breaks) - Alterations to workload (i.e. fewer tasks than normal within a time, more time to complete usual tasks and not to work tight deadlines) - Temporary changes to duties/tasks (i.e. alternative work/duties) - Support – someone to ask or check with, “buddy system,” time off for appointments, not working in isolation - Clear objectives and review mechanisms - Working from home part of the time where possible - Regular checks on whether the symptoms are fluctuating - Seek advice from Occupational Health/Human Resources – especially if is likely considered a disability   - Regular follow-ups to monitor progress from a biopsychosocial and occupational perspective  - support patients in adjusting their mental timeline of recovery to reflect a potentially prolonged course with a long phased RTW  - employers should discuss suitable adjustments with employees to aid their RTW  - both parties, employer and employee, should receive written advice  - referrals to occupational health services and medical reports are needed to support RTW process  - emphasize the relapsing-remitting nature of the illness to prevent premature RTW due to employer pressure  - the doctor with current clinical responsibility should complete a fit note  - RTW after illness is a marker of recovery  -clinicians should record work status in clinical notes for chronic ill health cases  - counting days lost to sickness and lost income due to Long COVID is essential from a public health perspective  **RTW recommendations essentially state:**   - Multidisciplinary treatment approach to address Long COVID symptoms impacting RTW - Policy changes for RTW that address disability benefits for Long COVID, or any pathway designed to protect employment - Actions need to be taken through policy adjustments for employment rights and sick pay - All countries need to review their benefit packages and access to disability benefits for Long COVID as there are only a few addressing this currently.   - Assess individuals using a holistic multidisciplinary approach and see how their life is impacted especially work  - Use shared decision making to discuss and agree on what support and rehabilitation will work for the individual with Long COVID  - create individualized rehabilitation programs addressing all problem areas, including RTW  - provide treatment for Long COVID symptoms that impact work  - continue to follow-up and monitor individuals even when they RTW and provide information for self-management and who to reach out to if there is a flare up  **Workplace accommodations:**   - Essential for patients with mild to moderate symptoms of Long COVID to stay functionally engaged - Adjustments to the job or work environment enable a safe and timely RTW   **Physician-Directed restrictions and limitations:**   - Restrictions: tasks a person can do but should avoid for medical reasons - Limitations: tasks a person is not currently capable of doing for medical reasons   **Typical RTW accommodations for fatigue:**   - Reduced work hours - Reduced work volume - Later start times to compensate for sleep disruption - Increased flexibility to self-pace workload - Avoidance of tight deadlines - Excusal from complex tasks with higher cognitive demands   **Importance of Physician guidance:**   - Ensures accommodations preserve remaining functionality - Protects against secondary health effects of worklessness |
